# Accelerometer data processing pipeline using ActiLife software for reproducible 24-hour physical behavior assessment in the ELSA-Brasil cohort

**DOI:** 10.1016/j.mex.2026.104092

**Published:** 2026-08-07

**Authors:** Bruno Pereira de Moura, Jeffer Eidi Sasaki, Rosane Harter Griep, Natan Feter, Maria Inês Schmidt, Maria de Jesus Mendes da Fonseca

**Affiliations:** a Oswaldo Cruz Foundation, Sérgio Arouca National School of Public Health, Graduate Program in Public Health Epidemiology, Rio de Janeiro, RJ, Brazil; b Federal University of Triângulo Mineiro, Department of Sport Sciences, Graduate Program in Physical Education, Uberaba, MG, Brazil; c Oswaldo Cruz Foundation, Oswaldo Cruz Institute, Laboratory of Education in Environment and Health, Graduate Program in Public Health Epidemiology, Rio de Janeiro, RJ, Brazil; d Federal University of Rio Grande do Sul, Postgraduate Program in Epidemiology, Porto Alegre, RS, Brazil

**Keywords:** Physical activity, Sedentary behavior, Sleep, Accelerometry, ActiGraph, Cohort studies, Reproducibility

## Abstract

This protocol describes a standardized and reproducible accelerometer data collection and processing workflow implemented in the ELSA-Brasil cohort using ActiLife software. The protocol enables consistent derivation of physical activity, sedentary behavior, and sleep metrics from hip-worn accelerometers in large epidemiological studies.

Protocol summary:

• Defines standardized procedures for accelerometer configuration, deployment, and data acquisition under 24-hour free-living conditions;

• Specifies predefined ActiLife processing parameters including wear-time validation, sleep detection, and activity intensity classification;

• Generates harmonized accelerometry-derived variables supporting replicable epidemiological analyses and cross-cohort comparability;

This protocol provides a parameter-explicit framework for accelerometer data processing using ActiLife software and may serve as a reference standard for cohort studies using similar accelerometry approaches.

## Graphical abstract

**Figure:**
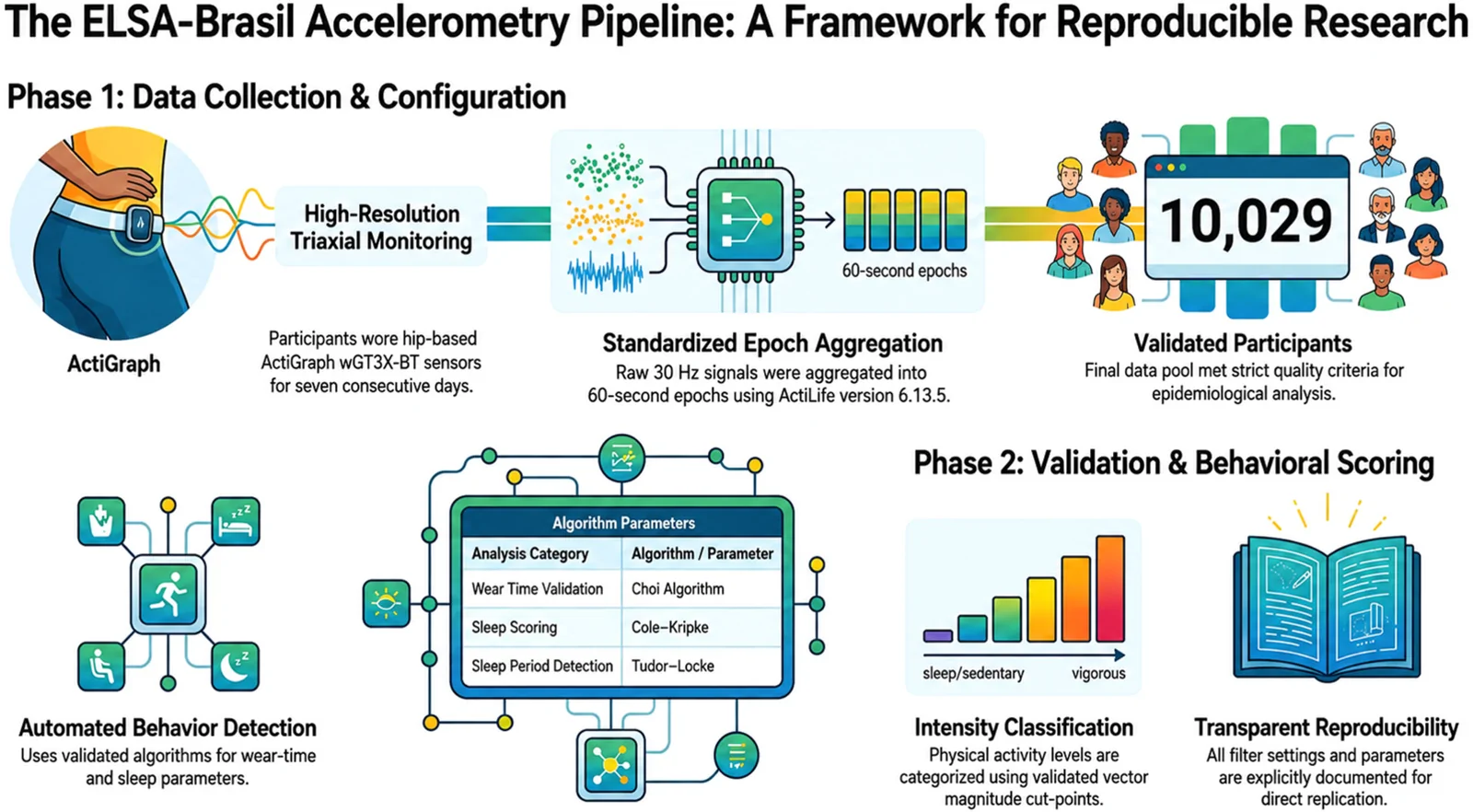

**Specifications table**

This table provides general information on your protocol.

**Table:** 

**Subject area**	Medicine and Dentistry
**More specific subject area**	Physical activity epidemiology and accelerometry data processing
**Name of your protocol**	Accelerometer data processing protocol using ActiLife: ELSA-Brasil cohort
**Reagents/tools**	ActiGraph wGT3X-BT accelerometers (Ametris LLC, Pensacola, FL, USA) ActiLife software version 6.13.5 Secure data storage server
**Experimental design**	24-hour hip-worn accelerometry protocol with standardized ActiLife processing workflow including wear-time validation, sleep detection, and activity classification
**Trial registration**	Not applicable
**Ethics**	This protocol was approved by the National Research Ethics Committee (CAAE: 56,021,516.0.1001.5240). All participants provided written informed consent.
**Value of the Protocol**	• Enables exact replication of accelerometer data processing using specified ActiLife parameter settings • Facilitates methodological harmonization across epidemiological cohorts using ActiGraph accelerometers • Improves reproducibility, transparency, and comparability of accelerometer-derived physical behavior metrics

## Background

Accelerometry provides device-based estimates of physical activity (PA), sedentary behavior (SB), and sleep that reduce reliance on self-reported information; however, these estimates are not independent of methodological decisions, and their reproducibility depends critically on explicit documentation of device configuration, software settings, signal processing parameters, and classification criteria [1,2]. Among the most commonly used accelerometers in epidemiological research, ActiGraph devices have gained prominence due to their ability to capture high-resolution movement data across different activity intensities [1,2]. These devices provide detailed assessments of movement intensity, duration, and patterns, enabling comprehensive evaluation of PA and SB across diverse populations [1]. Additionally, recent advancements in accelerometer technology have facilitated estimation of sleep parameters, further expanding their utility in behavioral and health research [3].

Despite growing harmonization efforts, accelerometer-derived behavioral estimates remain highly sensitive to methodological decisions, including epoch length, wear-time validation algorithms, sleep detection rules, and intensity classification thresholds. These parameter choices can substantially influence estimates of PA, SB, and sleep, limiting comparability between cohort studies and affecting reproducibility of epidemiological findings. Methodological variability in accelerometer data processing has been consistently identified as a major source of heterogeneity across epidemiological studies, emphasizing the need for transparent and well-documented processing protocols [1,4].

Several studies have proposed methodological frameworks to optimize accelerometer data processing, particularly in PA and SB research [1]. However, existing approaches vary widely, and consensus on best practices remains limited [1]. In large cohort studies, accelerometer implementation presents additional challenges, as participant heterogeneity, varied data collection conditions, and longitudinal designs may affect comparability and reproducibility [5]. Although initiatives such as the International Children’s Accelerometry Database (ICAD) and the Prospective Physical Activity, Sitting, and Sleep (ProPASS) consortium have advanced harmonization efforts, methodological variability persists, especially for adult and aging populations [6,7]. This ongoing variability reinforces the importance of clearly defined and standardized processing protocols to ensure methodological consistency across studies.

In this context, the variables reported in this study correspond to the complete set of metrics generated by the ActiLife software from accelerometer files, including PA summaries, sedentary bouts and breaks, and sleep parameters [1,2]. Additional metrics, such as activity energy expenditure, could not be estimated directly because these estimates require calibration equations and auxiliary parameters beyond accelerometer-derived counts [8,9]. Therefore, our approach was to document in detail the criteria and parameters applied in ActiLife, selected based on established literature [1], ensuring the generation of all available software-derived variables. By explicitly defining these procedures, this protocol enhances transparency, reproducibility, and comparability across studies using similar accelerometry approaches.

Within this methodological context, the Longitudinal Study of Adult Health (ELSA-Brasil) provides an opportunity to implement and document a standardized 24-hour accelerometry protocol in a large middle-aged and older adult population. The ELSA-Brasil is a multicenter cohort designed to investigate determinants of chronic diseases in Brazil [10,11]. In its third wave, accelerometry was incorporated to assess PA, SB, and sleep, highlighting the importance of standardized protocols. To our knowledge, no previous publication has provided a parameter-explicit description of the ActiLife processing pipeline implemented in this cohort. Therefore, this study describes a standardized and parameter-explicit accelerometer data collection and processing protocol using ActiLife software within the ELSA-Brasil cohort.

## Description of protocol

### Study context: the ELSA-Brasil multicenter cohort

The ELSA-Brasil is the largest ongoing multicenter epidemiological cohort in Latin America, investigating incidence, progression, and risk factors for diabetes and cardiovascular diseases among Brazilians [10,11]. Baseline data (2008–2010) were collected from 15,105 active and retired civil servants aged 35–74 years from six public institutions across different Brazilian states: Fundação Oswaldo Cruz (FIOCRUZ – Rio de Janeiro), University of São Paulo (USP), and the Federal Universities of Bahia (UFBA), Espírito Santo (UFES), Minas Gerais (UFMG), and Rio Grande do Sul (UFRGS).

The cohort is organized into six Research Centers located in the capital cities of Brazil’s Northeast, Southeast, and South regions, with follow-ups (waves) approximately every 4 years [11]. Exclusion criteria included severe cognitive or communication impairment, imminent departure from the institution (not due to retirement), and residence outside the corresponding metropolitan area for retirees. Women currently pregnant or recently delivered were rescheduled to ensure first interviews 4 months postpartum [11]. Recruitment combined advertisements, direct mail, phone calls, and a randomized employee list stratified by sex, age, and occupational category, ensuring balanced representation [11].

Ethical approval was obtained from all participating institutions’ Ethics Committees and the National Research Ethics Committee (CAAE: 56,021,516.0.1001.5240; approval number 1.900.315 dated January 27, 2017). All participants provided informed consent [12].

To facilitate reproducibility and clarity, Table 1 provides definitions of key terms related to accelerometer devices, data processing, and behavioral classification used throughout this protocol. The terms are grouped thematically to reflect the logical sequence of accelerometer data handling, from device specifications and raw data features through processing steps to the classification of PA, SB, and sleep.

**Table 1:** Definitions of key terms used in accelerometer data processing and analysis.

Term	Definition
Accelerometer [2]	A small wearable device that measures acceleration forces to objectively assess movement patterns in three-dimensional space; in this study, the device was worn on the hip.
Sensors [1]	Components within accelerometers (e.g., piezoelectric, piezoresistive, capacitive) that detect acceleration signals.
Micro-electro-mechanical systems (MEMS) [1]	Miniaturized mechanical and electrical components used in modern accelerometers for high-precision motion detection.
Inclinometer [2]	A sensor that detects body posture or orientation (e.g., standing, sitting, lying) based on device angle relative to gravity.
Uniaxial, biaxial, triaxial [2]	Refers to accelerometers measuring acceleration along one, two, or three orthogonal axes, respectively.
Vector magnitude (VM) [1]	A composite measure of acceleration combining three axes into a single magnitude (√ [x² + y² + z²]) to represent overall movement intensity.
Sampling frequency [1]	The frequency (Hz) at which raw acceleration data are recorded per second by the accelerometer.
Raw data [1]	Unprocessed acceleration signals recorded by the accelerometer sensors before any data transformation or aggregation.
Filter [1]	Signal processing step to remove noise or artifacts from raw accelerometer data before analysis.
Epoch [2]	A predefined time window (e.g., 60 s) over which raw data are summarized into counts or other metrics.
Bout [2]	A sustained period of physical activity or sedentary behavior meeting specified minimum duration criteria.
Counts [1]	Unitless values derived from raw acceleration data representing the intensity and frequency of movement within a specified epoch.
Wear time validation [13,14]	The process or algorithm used to identify and confirm valid wear periods from raw accelerometer data, distinguishing wear from non-wear time.
Cut-points [1]	Threshold values of counts per minute used to classify intensity levels of physical activity (e.g., light, moderate, vigorous), sedentary behavior, and sleep time.
Physical behaviors [15]	Observable patterns of human movement and posture, including physical activity, sedentary behavior, and sleep, which together comprise daily movement profiles.
Physical activity (PA) [16]	Any bodily action produced by the contraction of skeletal muscles resulting in an increase in energy expenditure above resting levels (> 1.5 METs).
Sedentary behavior (SB) [16]	Waking behavior characterized by energy expenditure ≤1.5 METs while sitting or reclining.
Activity energy expenditure [9]	The portion of total energy expenditure attributable to energy used during physical activities above resting metabolic rate, including the caloric cost of both exercise and non-exercise activity thermogenesis.
Metabolic equivalent of task (MET) [9]	A standardized unit estimating the energy cost of physical activities, where 1 MET represents the energy expenditure at rest (1 MET ≈ 1 kcal per kilogram of body weight per hour).

Note: The terms are organized thematically to reflect the logical progression of accelerometer data handling, from device characteristics and raw data features to data processing steps and classification of physical behaviors. This organization facilitates a clear understanding of methodological concepts employed in the study.

### Accelerometer deployment and data collection protocol in ELSA-Brasil

Accelerometry data collection was first implemented in ELSA-Brasil during the third follow-up wave (2017–2019) and has since been incorporated into subsequent waves. Among the 12,636 participants eligible for the third wave, 10,335 agreed to wear accelerometers. Of these, 46 either did not return the device or withdrew, resulting in 10,289 participants who completed the protocol.

Physical behaviors were assessed using the ActiGraph wGT3X-BT® (Ametris LLC, Pensacola, FL, USA), one of the most widely used accelerometer models in epidemiological research. The compact device contains a triaxial micro-electro-mechanical systems (MEMS) accelerometer capable of measuring acceleration along three orthogonal axes within a dynamic range of ±8 g at sampling frequencies between 30 and 100 Hz. Raw acceleration signals can be analyzed individually by axis or combined into vector magnitude, calculated as the square root of summed squared accelerations (√[x² + y² + z²]) [17]. The device includes an ambient light sensor and inclinometer-based posture estimation capability; however, it does not include a direct wear-time detection sensor. Wear time is instead determined algorithmically based on movement patterns using validated wear-time classification algorithms such as the Choi algorithm [1,13]. For use in Brazil, the device is registered with the Brazilian Telecommunications Agency (ANATEL, homologation number 06,586-19–09,607).

In ELSA-Brasil, raw triaxial acceleration was sampled at 30 Hz for each participant and subsequently processed in ActiLife® (version 6.13.5). Raw data files were converted to 60-second epoch datasets, and activity counts were generated using validated algorithms to produce standardized output metrics (see Section “Accelerometer data processing pipeline using ActiLife software” for details) [18,19].

Participants wore the device on the right hip, aligned with the anterior axillary line and iliac crest, secured by an elastic belt for 7 consecutive days (24 h/day), removing it only for water-based activities (e.g., bathing or swimming) [1]. Detailed instructions were provided on device wear and completion of the sleep diary [20]. Devices were programmed in ActiLife on the day prior to baseline visits, including battery check and calibration [20].

Research staff distributed devices after baseline assessments at the end of the Wave 3 clinical assessment visit (Day 1). Trained research assistants hand-delivered the accelerometers to participants, rather than using a mail-order strategy. Research assistants provided standardized in-person instructions on device use, including how to adjust the waistband, position the device above the right anterior superior iliac spine, remove it only during water-based activities, and complete the sleep diary. Participants left the study center wearing the device. Additional support was available during business hours, and research assistants conducted a follow-up phone call on day 2 to encourage compliance and address questions. Participants were also contacted before the second in-person appointment, scheduled approximately 9 to 12 days after the first visit, to remind them to return the accelerometer. At this return visit, research assistants collected the accelerometers, downloaded the .gt3x files, labeled them by study ID, and verified sleep diary completeness [20].

Following data download, wear time was evaluated using the Choi algorithm to assess compliance with the monitoring protocol [20]. Participants not meeting a minimum wear-time threshold of 60% of the 7-day monitoring period (100.8 of 168 h) were invited to repeat the accelerometer protocol to ensure adequate data collection quality [20]. This criterion was applied as part of the field-level data collection quality control procedures and does not correspond to the analytical inclusion criteria applied during accelerometer data processing.

Participants received individualized reports, including step counts and moderate-to-vigorous PA (MVPA, classified using the Freedson algorithm [9]), at device return and via email [20]. Data files (.gt3x, approximately 40–50 MB each) were uploaded to a secure server at the Universidade Federal do Rio Grande do Sul, ensuring centralized management and access for analysis [20].

### Accelerometer data processing pipeline using actilife software

The processing criteria implemented in ActiLife for this study are not standardized across the field. They represent evidence-based selections aligned with current recommendations and consensus statements, tailored to the age distribution, wear location, and 24-h protocol used in ELSA-Brasil. Where multiple acceptable parameters exist (e.g., epoch length, non-wear definitions, sleep scoring/detection), we chose parameters with empirical support and operational feasibility for this cohort. All parameterization steps are reported in detail to facilitate replication and clarify implications for between-study comparability [1,15].

### Software configuration and reproducibility

All ActiLife processing settings were saved and archived using ActiLife configuration files and standardized batch-processing parameters. The following settings were explicitly specified: epoch length, filter configuration, wear-time validation parameters, sleep scoring and sleep period detection algorithms, bout definitions, vector magnitude cut-point thresholds, and output variable selection. Use of a fixed ActiLife software version (6.13.5) and standardized configuration ensured that all derived accelerometry variables can be reproduced exactly. Configuration files, batch-processing templates, and software settings were archived and are available upon request to support replication of the processing workflow. To further support reproducibility, Supplementary File 1 provides screenshots of the ActiLife processing settings used in this protocol, including epoch conversion, filter configuration, wear-time validation, sleep analysis, and activity classification settings.

### Accelerometer data processing workflow sequence

**Fig. 1:**
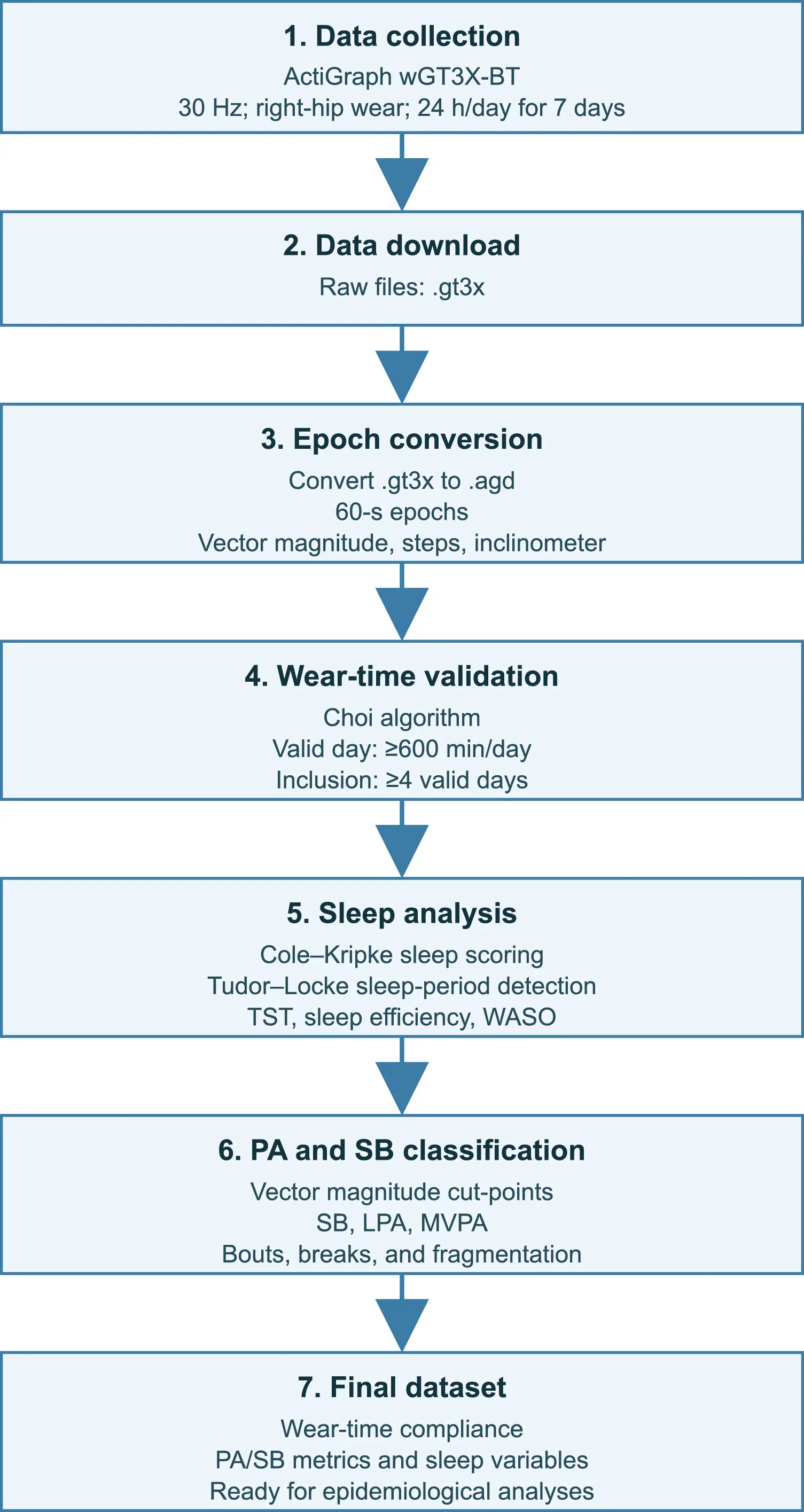
Accelerometer data processing workflow in ELSA-Brasil. The flowchart summarizes the ActiLife-based processing pipeline from raw accelerometer data acquisition to generation of the final analytical dataset, including epoch conversion, wear-time validation, sleep analysis, and physical activity/sedentary behavior classification. Abbreviations: .agd, ActiGraph epoch-level data file; .gt3x, ActiGraph raw accelerometer data file; LPA, light-intensity physical activity; MVPA, moderate-to-vigorous physical activity; PA, physical activity; SB, sedentary behavior; TST, total sleep time; WASO, wake after sleep onset.

The accelerometer data processing pipeline was implemented in a standardized and transparent workflow. The complete pipeline, including data collection, wear-time validation, sleep analysis, and behavioral classification, is illustrated in Fig. 1, enabling visualization of the operational workflow and facilitating replication. The detailed parameter-specific implementation steps applied in ActiLife are described below to ensure consistent generation of accelerometer-derived variables.

The processing steps were applied as follows:

1. Acquire raw accelerometer data (.gt3x format) sampled at 30 Hz using ActiGraph wGT3X-BT devices
2. Import raw files into ActiLife version 6.13.5
3. Convert raw acceleration signals into epoch-level count files (.agd format) using the following parameters:
  • Epoch length: 60 s
  • Filter: Normal filter enabled
  • Low-frequency extension: Disabled
4. Apply wear-time validation using the Choi algorithm with the following parameters:
  • Minimum non-wear duration: 90 min
  • Spike tolerance: 2 min
  • Upstream/downstream window: 30 min
5. Execute sleep detection using the following configuration:
  • Sleep scoring algorithm: Cole–Kripke
  • Sleep period detection algorithm: Tudor–Locke
6. Classify waking physical behaviors using validated vector magnitude cut-points
7. Generate output variables including activity counts, bout metrics, SB metrics, and sleep parameters

To facilitate use of this protocol as a methodological reference, Table 2 summarizes the main processing decisions, selected parameters, rationale, and key considerations for adaptation to other populations or research questions.

**Table 2:** Summary of key accelerometer processing decisions, rationale, and considerations for adaptation.

Processing decision	Parameter adopted in ELSA-Brasil	Rationale	Key considerations for adaptation
Device and raw data acquisition	ActiGraph wGT3X-BT; raw triaxial acceleration sampled at 30 Hz; right-hip placement; 24-hour protocol for 7 consecutive days.	This configuration supports integrated assessment of PA, SB, and sleep using a single device and maintains continuity with the historical ActiGraph literature on hip-worn accelerometry [1,5,15,17]	Different wear locations, such as wrist placement, may improve some sleep-related estimates but reduce direct comparability with hip-worn PA and SB cut-point approaches [15,18,19,21,22]
Epoch length	Raw .gt3x files converted to 60-second epoch-level .agd files.	The 60-second epoch preserves consistency with the validation protocol of the selected vector magnitude cut-points, which were developed using count-per-minute outputs and 60-second aggregation [1,18,19]. It also represents a practical compromise between temporal resolution, computational efficiency, and comparability in large cohorts [1,5]	Shorter epochs may better capture short or intermittent activity bouts but can reduce comparability with cut-points validated using 60-second aggregation [1,4,15]. Future analyses requiring shorter epochs should return to the retained raw .gt3x files and apply appropriately validated thresholds or raw-data methods [23]
Filter configuration	Normal filter enabled; Low Frequency Extension disabled.	The Normal filter was retained to preserve consistency with the selected cut-point framework and to avoid filter-dependent changes in ActiGraph count generation [8,19]	The Low Frequency Extension may increase sensitivity to low-amplitude movement but can alter estimated steps and time classified as SB, light-intensity PA, and MVPA [24,25]. It may be appropriate for populations with very slow movement patterns, but only when thresholds validated under that filtering condition are available.
ActiLife software version and count generation	ActiLife version 6.13.5; standardized signal-processing pipeline including filtering, rectification, thresholding, and epoch summation.	Using a fixed software version and standardized settings supports reproducibility within the ActiLife environment [8]	ActiLife-derived counts are processed transformations of raw acceleration signals and may not be exactly reproducible across independent software platforms or different software versions [8,26]
Wear-time validation algorithm	Choi algorithm using vector magnitude; 90 min of consecutive zero counts; 2-minute spike tolerance; 30-minute upstream/downstream window.	The Choi algorithm is a validated automated approach for distinguishing wear and non-wear time in adults, supporting standardized data inclusion [13,14]	Automated wear-time algorithms reduce reliance on self-reported logs but may still misclassify prolonged inactivity or atypical movement patterns [27]. Alternative algorithms may influence valid wear time and sample inclusion.
Valid-day criteria	Valid day defined as ≥600 min of wear time; at least four valid days required, including two weekdays and one weekend day.	These criteria provide a balance between data quality, participant retention, and comparability with commonly used accelerometry processing recommendations [1]	More restrictive criteria may improve stability of estimates but reduce sample size and representativeness. More permissive criteria may increase inclusion but reduce reliability of average daily estimates [1,5]
Sleep scoring algorithm	Cole–Kripke algorithm.	The Cole–Kripke algorithm is an established sleep/wake scoring algorithm with demonstrated sensitivity for detecting sleep in adult populations [3,28]	The algorithm was originally developed for wrist actigraphy; its application to hip-worn accelerometry is a pragmatic extension and may affect estimates of sleep duration, awakenings, and fragmentation [21,28]
Sleep-period detection algorithm	Tudor–Locke algorithm, default mode, used to identify the principal sleep period.	The Tudor–Locke algorithm was developed for waist-worn accelerometry to distinguish the main sleep-period time from waking physical activity and sedentary behavior in 24-hour protocols [29]	This approach identifies one principal nocturnal sleep episode. Daytime naps are not detected as separate sleep periods and may be classified as sedentary behavior [29,30]
Order of sleep and waking behavior processing	Sleep processed before PA and SB classification.	Processing sleep first reduces the likelihood that sleep periods are misclassified as waking SB or PA, supporting a more coherent 24-hour behavioral framework [1,15]	Different processing sequences or diary-assisted sleep definitions may change estimates of waking wear time, SB, and PA.
Acceleration metric for PA and SB classification	Vector magnitude counts derived from the three orthogonal axes.	Vector magnitude incorporates acceleration from all three axes and is compatible with the selected cut-points [[17], [18], [19]]	Vector magnitude count-based outputs are not directly comparable with vertical-axis-only methods or raw acceleration metrics such as ENMO [4,8,15,31,32]
Sedentary behavior cut-point	SB defined as <200 counts per minute using vector magnitude.	This threshold was selected based on the cut-point proposed by Aguilar-Farías et al., which showed favorable classification performance for sedentary behavior in older adults [18]	SB estimates may differ when using alternative thresholds, device placements, axes, or raw acceleration approaches [1,4,15]
Light-intensity PA and MVPA cut-points	LPA defined as ≥200 and <2751 counts per minute; MVPA defined as ≥2751 counts per minute.	These thresholds were selected because they were derived from vector magnitude counts, were compatible with 60-second epoch aggregation, and were appropriate for the cohort’s age profile and available validation evidence [1,19]	The MVPA threshold has limited specificity, so MVPA estimates should be interpreted cautiously, especially when comparing results across studies using different cut-points or processing pipelines [1,19]
Moderate and vigorous activity aggregation	Moderate and vigorous intensities aggregated as MVPA.	This approach aligns with public health guidelines that operationalize recommendations using combined MVPA [16,33]. Vigorous PA is typically minimal in large adult cohorts [34,35]	Separate vigorous-intensity analyses may be unstable in populations with very low vigorous activity accumulation.
Bout definition	Sustained activity bouts identified using a minimum duration of 1 min and a drop time of 0 min.	This definition provides fine-grained bout metrics and is consistent with contemporary approaches that do not require longer minimum bout durations for health-related PA interpretation [15,16,33]	Alternative bout definitions, such as 5- or 10-minute bouts or different drop-time allowances, can substantially alter accumulated bout-based PA and SB estimates.
Use of participant logs in classification	Participant activity logs were not used for PA and SB classification.	Automated classification reduces reliance on self-reported logs and supports standardized processing across a large cohort [1,27,30]	Logs may provide contextual information but can introduce missingness, reporting error, and additional participant burden.
Daily time window and averaging	All days analyzed from 00:00 to 23:59; average daily time calculated as the mean across all valid days.	This approach provides standardized daily estimates of time spent in each behavioral category and facilitates comparison across participants.	Different averaging strategies or weighting of weekdays and weekend days may influence estimates of habitual behavior.
Future compatibility with raw acceleration approaches	Raw .gt3x files retained and centrally stored; ActiLife-derived variables generated as a reproducible count-based dataset.	This preserves compatibility with historical ActiGraph count-based literature while allowing future raw acceleration processing pipelines, including GGIR-based approaches [4,8,15,31,32]	Raw acceleration methods may improve transparency and cross-device harmonization but require additional programming expertise, computational resources, and explicit documentation of analytical decisions [5,31,32]

Note: This table summarizes the main processing decisions adopted in the ELSA-Brasil ActiLife-based accelerometer data processing protocol. The rationale reflects the cohort’s age profile, hip-worn 24-hour monitoring design, selected ActiGraph count-based outputs, and compatibility with the validation studies underlying the chosen algorithms and cut-points. These decisions should not be interpreted as universal recommendations. Adaptations to other populations, devices, wear locations, software platforms, or research questions should be explicitly justified and documented. Bracketed numbers refer to the manuscript reference list. Abbreviations: .agd, ActiGraph epoch-level data file; .gt3x, ActiGraph raw accelerometer data file; ActiLife, ActiGraph software; ENMO, Euclidean Norm Minus One; GGIR, an open-source R package for processing raw accelerometer data; LPA, light-intensity physical activity; MVPA, moderate-to-vigorous physical activity; PA, physical activity; SB, sedentary behavior.

### Conversion of raw accelerometer data to epoch format

Raw triaxial acceleration data (.gt3x files) recorded at 30 Hz were converted to epoch-level data (.agd files) using the “Import/Export/Convert” functionality of ActiLife software version 6.13.5, with a 60-second epoch length, steps and inclinometer data retained, the Normal filter enabled, and Low Frequency Extension disabled. Filter configuration influences activity count generation and was standardized across all participants to ensure internal consistency.

The 60-second epoch was selected primarily to preserve consistency with the validation protocol of the vector magnitude cut-points used in this study, which were developed using count-per-minute outputs and applied as 60-second aggregated thresholds [18,19]. Although shorter epochs, such as 5 or 15 s, may provide greater temporal resolution and may better capture short or intermittent activity bouts, epoch length is a known source of variability in accelerometer-derived estimates, and changing epoch duration may affect comparability across studies and with calibration-specific cut-points [1,4,15]. In large epidemiological cohorts, 60-second epochs also represent a practical compromise between temporal resolution, computational efficiency, and comparability with commonly used count-based processing approaches [1,5]. Because reintegration of .agd files in ActiLife is limited to larger epoch periods, future analyses requiring shorter epochs would need to return to the retained raw .gt3x files and reconvert them using the desired epoch length [23]. This preserves the possibility of future reanalysis using alternative epoch settings, provided that the raw accelerometer files remain available and that any new epoch length is matched to appropriately validated cut-points or analytical methods.

Activity counts were generated using ActiLife’s standardized signal-processing pipeline, including filtering, rectification, thresholding, and epoch summation [8]. Although the ActiLife count-generation process has been described in the literature, exact reproduction across independent software platforms cannot be fully guaranteed due to implementation-specific processing steps [8]. However, reproducibility within the ActiLife environment is ensured using identical software version, filter configuration, and processing parameters.

The Normal filter was selected to preserve consistency with the processing conditions underlying the selected vector magnitude cut-points and to avoid introducing filter-dependent changes in ActiGraph count generation [8,19]. This decision is important because ActiGraph activity counts are processed transformations of acceleration signals and may vary according to software-specific filtering procedures [8,26]. The Low Frequency Extension increases sensitivity to lower-amplitude movement and may therefore be useful in studies focused on low-intensity activity patterns or populations with very slow movement; however, studies comparing the Normal filter and the Low Frequency Extension have shown that enabling this option can substantially alter estimated steps and time classified as sedentary behavior, light-intensity PA, and MVPA [24,25]. Therefore, enabling the Low Frequency Extension in the present protocol could have reduced comparability with the selected calibration thresholds and potentially shifted participants across intensity categories, particularly because the selected cut-points were not validated under that filtering condition [15,19]. For this reason, the Normal filter was retained and applied uniformly across all participants to support internal consistency and comparability with the selected cut-point framework [8,19].

### Wear time validation

Wear and non-wear periods were identified using the Choi algorithm implemented within ActiLife’s “Wear Time Validation” tab [13,14]. This method flags non-wear based on 90 consecutive minutes of zero counts, allowing up to 2 min of spike tolerance within 30-minute upstream/downstream windows on the vector magnitude [13,14]. A validation study conducted in adults reported 100% sensitivity and 99.9% specificity [13,14]. Vector magnitude was selected as the primary axis input, consistent with the original Choi algorithm implementation [1,13].

A valid day was defined as ≥600 min of wear time, with at least four valid days (including two weekdays and one weekend day) required for inclusion in further analyses [1]. Optimal Screen Parameters were set to include all wear periods, and sleep periods were marked as wear time.

### Sleep analysis

Sleep was analyzed with the Batch Sleep function in ActiLife, combining the Cole-Kripke sleep scoring algorithm [28], and the Tudor-Locke sleep period detection method [29] a combination validated for use in free-living epidemiological studies [3,21]. Default detection parameters were used: minimum sleep period of 160 min, maximum of 1440 min, and minimum of 15 non-zero epochs. Bedtime was defined as the first occurrence of ≥5 consecutive minutes scored as sleep, and wake time as the first occurrence of ≥10 consecutive minutes scored as wake following a sleep period [29]. To reduce misclassification of behaviors, sleep was processed prior to the classification of PA and SB so that waking behaviors were only identified outside periods designated as sleep [1,15].

The Cole–Kripke algorithm was selected for sleep scoring due to its demonstrated sensitivity for detecting sleep in adult populations compared with alternative scoring algorithms [3,28]. However, because this algorithm was originally validated using wrist-worn actigraphy, its application to hip-worn accelerometers represents a pragmatic extension and may result in systematic overestimation of sleep duration and reduced sensitivity for detecting nocturnal awakenings [1,21]. This limitation reflects the absence of sleep-scoring algorithms specifically validated for hip-worn accelerometry and should be considered when interpreting sleep-derived variables.

Although wrist placement has become more common in sleep-focused protocols, hip placement remains advantageous for consistency with historical PA literature and allows integrated 24-hour behavior profiling using a single anatomical site [15]. Evidence indicates that hip-worn implementations yield reasonable estimates of total sleep time (TST) [36,37], although they may overestimate sleep duration and show reduced sensitivity to short awakenings or sleep fragmentation relative to PSG [3,21]. These findings support the feasibility of this approach while reinforcing the need for cautious interpretation of sleep continuity metrics.

For sleep period detection, the Tudor–Locke algorithm was applied in its default mode [29]. Importantly, this method is designed to identify one main nocturnal sleep period per 24-hour cycle (i.e., a consolidated nighttime episode between algorithm-defined bedtime and wake time) [29]. Shorter daytime sleep episodes (e.g., naps) are not detected by the sleep analysis algorithm and were therefore quantified as periods of SB in subsequent analysis. Thus, the TST derived in ELSA-Brasil reflects the nocturnal sleep episode only [29].

This analytic strategy aligns with contemporary 24-hour accelerometry protocols in large epidemiological cohorts (e.g., UK Biobank, Whitehall II), which rely on continuous monitoring and standardized algorithms to delineate the principal sleep episode in free-living populations [31,38,39]. While this enhances ecological validity and supports reproducibility [1,39], differences in wear location (hip versus wrist) and algorithmic rules should be considered when interpreting sensitivity to brief awakenings and sleep continuity [1,21,26].

### Physical activity and sedentary behavior classification

We analyzed SB and PA using vector magnitude data derived from raw acceleration signals recorded along three orthogonal axes, with activity intensities classified using cut-points validated in older adult populations [18,19]. These vector magnitude cut-points were applied due to the cohort’s age distribution and the absence of cohort-specific calibration studies. They were selected because, among the cut-points available at the time of analysis, they showed favorable performance regarding error rates, sensitivity, and specificity [18,19]. This conservative classification approach reduces the likelihood of overestimating moderate-to-vigorous PA. However, because count-based cut-points are population- and device-specific, their application outside the original calibration population may introduce classification bias [1,4].

Specifically, SB was defined as activity below 200 counts per minute (cpm), based on the cut-point proposed by Aguilar-Farías et al., which demonstrated a correct classification rate of 86.3%, with sensitivity of 87.8% and specificity of 83.7% [18]. Light-intensity PA (LPA) was defined as ≥200 and <2751 cpm, while MVPA was defined as ≥2751 cpm, according to Santos-Lozano et al., [19]. an approach consistent with recent systematic reviews and current recommendations for accelerometer data harmonization [1]. Alternative calibration approaches were considered; however, these thresholds were retained because they were derived from vector magnitude counts, were compatible with 60-second epoch aggregation, and provided an appropriate balance between methodological consistency, cohort age profile, and classification performance. The MVPA threshold showed a sensitivity of 72.5% and a specificity of 31.5%, indicating that MVPA estimates should be interpreted cautiously, particularly when comparing results with studies using different calibration methods or processing pipelines [1,19]. Although this study identified a separate threshold for vigorous PA (≥9359 cpm), time accumulated at this intensity is typically minimal in large adult cohorts, generally representing <1% of daily wear time [34,35].

Consistent with current public health guidelines, which operationalize activity recommendations in terms of combined MVPA [16,33], we therefore aggregated moderate and vigorous intensities under MVPA. SB and PA intensity cut-points and their validation metrics, including sensitivity and specificity, are summarized in Table 3 for ease of reference.

**Table 3:** Validated accelerometer cut-points for classification of physical behaviors with sensitivity and specificity metrics.

Physical behavior	Cut-point (VM cpm)	Sensitivity (%)	Specificity (%)	AUC	Reference
Sedentary behavior (SB)	< 200	87.8	83.7	0.86	Aguilar-Farías [18]
Light physical activity (LPA)	200 – 2750	68.5	27.5	0.70	Santos-Lozano [19]
Moderate-to-vigorous physical activity (MVPA)	≥ 2751	72.5	31.5	0.70	Santos-Lozano [19]

Note: Cut-points were applied to vector magnitude (VM) counts per minute derived from triaxial acceleration data. These cut-points were validated in older adult populations using hip-worn ActiGraph devices. Sensitivity, specificity, and area under the receiver operating characteristic curve (AUC) values correspond to the validation studies cited in the reference list.

Sustained activity bouts were identified using a minimum duration of 1 min and a drop time of 0 min [15,16]. Thresholds for total PA and MVPA were set at ≥200 cpm and ≥2751 cpm, respectively [18,19]. Non-wear periods were excluded based on the Choi algorithm, as described previously (see Section “Wear time validation”) [13,14]. Data from participant activity logs were not used in classification [1,30].

In ActiLife software, under “Global Date and Time Filters,” analysis included all days from 00:00 to 23:59. Average daily time (minutes per day) spent in each behavioral category was calculated as the mean across all valid days for each participant.

### Implications of methodological choices and adaptation to other populations

The methodological choices described in this protocol were selected to support transparency, internal consistency, and comparability with the validation studies underlying the selected ActiLife outputs [1,2,15]. These decisions involve trade-offs that should be considered when adapting the protocol to other populations or research questions. The use of 60-second epochs improves comparability with widely used count-based calibration studies and reduces computational burden in large cohorts [1,5,19], but it may smooth short bursts of movement and underestimate intermittent activity patterns that could be better captured using shorter epochs [1,4,15]. Conversely, shorter epochs may increase temporal resolution but can reduce direct comparability with cut-points validated using 60-second aggregation [1,19].

Filter configuration may also affect activity estimates because ActiGraph counts are processed transformations of raw acceleration signals and depend on software-specific filtering and count-generation procedures [8,26]. The Normal filter was retained to preserve consistency with the selected calibration thresholds and to avoid changing the count-generation process across participants [8,19]. Use of the Low Frequency Extension may increase sensitivity to lower-amplitude movements and can substantially alter estimated steps and time classified as sedentary behavior, light-intensity PA, or MVPA [24]. Therefore, although the Low Frequency Extension may be useful in populations characterized by very low-amplitude movement patterns, it may also shift participants across intensity categories when cut-points were not validated under that filtering condition [8,24]. Similarly, the selected wear-time algorithm and valid-day criteria support standardized data inclusion [13,14], although more permissive or restrictive criteria may influence sample size, participant representativeness, and estimates of average daily behavior [1,5,27].

Sleep-related estimates are also dependent on algorithmic decisions. In this protocol, the Cole–Kripke algorithm was used for sleep/wake scoring and the Tudor–Locke algorithm was used to identify the principal sleep period; however, these two components should be interpreted differently. The Cole–Kripke algorithm was originally developed for wrist actigraphy, whereas its application to hip-worn accelerometer data represents a pragmatic extension rather than a placement-specific validation [21,28]. In contrast, the Tudor–Locke algorithm was developed for waist-worn accelerometry to distinguish the main sleep-period time from waking physical activity and sedentary behavior [29]. This combined ActiLife-based approach provides a standardized method for identifying the principal nocturnal sleep episode in a 24-hour hip-worn protocol, but sleep estimates derived from this workflow may differ from estimates obtained using wrist-worn devices, diary-assisted protocols, polysomnography, or alternative sleep algorithms [3,21,29,30]. Accordingly, total sleep time, wake after sleep onset, and sleep fragmentation should be interpreted with caution, because sleep estimates may vary by wear location and algorithm, with hip-worn accelerometry showing limitations for some sleep parameters when compared with polysomnography and wrist-worn actigraphy [3,21,22].

Future compatibility with raw acceleration approaches should also be considered. Count-based ActiLife processing remains useful in settings where continuity with historical ActiGraph literature, compatibility with established cut-points, standardized graphical/software interfaces, and lower programming requirements are important [1,8,19]. At the same time, raw acceleration metrics, such as Euclidean Norm Minus One (ENMO), and open-source tools, such as GGIR, are increasingly used because they can improve transparency, facilitate cross-device harmonization, and support flexible processing of physical activity, sedentary behavior, and sleep outcomes [15,32]. These approaches, however, may require greater familiarity with accelerometry methods, programming skills in R or related environments, and computational resources for large-scale datasets [5,31,32]. Thus, the present ActiLife-based protocol should be viewed as complementary to raw acceleration pipelines rather than as a competing approach. This is particularly relevant for ELSA-Brasil, because previous analyses of the cohort’s accelerometry data have already used raw acceleration processing with GGIR to describe movement behaviors in middle-aged and older adults [20]. The ActiLife-derived database described here therefore complements this earlier work by documenting a parameter-explicit, count-based processing pipeline and generating a parallel set of reproducible variables within the ActiLife environment. Because raw .gt3x files were retained and centrally stored in ELSA-Brasil, future analyses may develop and document additional raw acceleration processing workflows, including GGIR-based pipelines, allowing comparison between count-based and raw-metric outputs and expanding the analytical value of the cohort.

## Data privacy and security

Ethical procedures related to ELSA-Brasil[12], including approval by institutional and national Ethics Committees and informed consent, are described in the section “Study context: The ELSA-Brasil multicenter cohort.” In addition, all participant data were securely stored and processed on servers fully compliant with applicable data protection standards [20]. Personally identifiable information was transformed into a unique anonymized participant identifier (IDELSA) [11].

## Protocol validation

Application of the accelerometer data processing pipeline to 10,289 raw accelerometer files resulted in 10,029 valid processed datasets following predefined wear-time, sleep detection, and activity classification criteria. This high proportion of valid datasets demonstrates the feasibility and operational robustness of the protocol in a large population-based cohort under free-living conditions.

Following implementation of the standardized processing criteria described above, ActiLife software generated a multidimensional analytical dataset describing PA, SB, and sleep across the 24-hour cycle. Processing was performed using literature-based methodological criteria for epoch conversion, wear-time validation, and behavioral classification, ensuring consistency with established accelerometry processing frameworks [1,8]. The resulting dataset comprised variables grouped into seven predefined categories: wear-time compliance, activity and sedentary bouts, sedentary breaks, physical behavior summaries, axis-specific acceleration counts, sleep parameters, and additional data formats, as summarized in Table 4. In addition to the primary SB definition based on <200 counts per minute using vector magnitude, the dataset includes an alternative SB metric based on <100 counts per minute (vector magnitude), derived upon request from investigators and incorporated as an additional analytical variable.

**Table 4:** Summary of derived variables from ActiLife-processed accelerometry data in the third wave of ELSA-Brasil.

Variable name	Definition	Unit
Wear time^a^^,^^g^	Mean daily accelerometer wear time.	min
Non-wear time^a^^,^^g^	Mean daily accelerometer non-wear time.	min
Wear days^a^	Total number of valid accelerometer wear days.	days
MVPA bouts^b^^,^^g^	Average number of daily moderate-to-vigorous physical activity (MVPA) bouts.	bouts/day
MVPA time bouts^b^^,^^g^	Average time of each MVPA bout.	min/bout
Physical activity (PA) bouts^b^^,^^g^	Average number of daily total PA bouts.	bouts/day
PA time bouts^b^^,^^g^	Average time of each total PA bout.	min/bout
Sedentary bouts^b^^,^^g^	Average number of daily sedentary bouts.	bouts/day
Sedentary time bouts^b^^,^^g^	Average time of each sedentary bout.	min/bout
Sedentary breaks^c^^,^^g^	Average number of daily sedentary breaks.	breaks/day
Total time sedentary breaks^c^^,^^g^	Average total time of daily sedentary breaks.	min/day
Sedentary breaks time^c^^,^^g^	Average time of each sedentary break.	min/break
Total sleep time (TST)^d^^,^^g^	Average daily total sleep time.	min/day
Sedentary behavior (SB)^d^^,^^g^	Average daily time of SB.	min/day
Light PA (LPA)^d^^,^^g^	Average daily time of LPA.	min/day
Moderate PA (MPA)^d^^,^^g^	Average daily time of MPA.	min/day
Vigorous PA (VPA)^d^^,^^g^	Average daily time of VPA.	min/day
MVPA^d^^,^^g^	Average daily time of MVPA.	min/day
Step counts^d^	Average daily step count.	steps/day
Steps per minute^d^	Average number of steps per minute.	steps/min
Fragmented physical activity (FPA)^d^^,^^g^	Ratio of the number of physical activity bouts to the total time of physical activity.	bouts/min
Axis 1 counts^e^	Average daily counts for axis 1 (y-axis).	counts/day
Axis 2 counts^e^	Average daily counts for axis 2 (x-axis).	counts/day
Axis 3 counts^e^	Average daily counts for axis 3 (z-axis).	counts/day
Axis 1 cpm^e^	Average counts per minute (cpm) recorded along axis 1 (y-axis).	cpm
Axis 2 cpm^e^	Average cpm recorded along axis 2 (x-axis).	cpm
Axis 3 cpm^e^	Average cpm recorded along axis 3 (z-axis).	cpm
Vector magnitude (VM) counts^e^	Average daily VM counts across all three axes.	counts/day
VM cpm^e^	Vector magnitude counts per minute.	cpm
Number of sleep periods (NSP)^f^	Average number of sleep bouts.	bouts/day
Sleep efficiency (SE)^f^	Sleep time divided by the total time the subject was in bed.	%
Sleep onset (SO)^f^	Clock time of sleep onset identified by the algorithm.	hour
Wake after sleep onset (WASO)^f^	Average daily total time recorded as awake after sleep onset.	min/day
Sleep activity counts (SAC)^f^	Average daily counts recorded during the entire sleep period.	counts/day
Sleep fragmentation index (SFI)^f^	Index of restlessness during the sleep period; higher values indicate greater sleep disruption.	%

a wear time compliance metrics.

b activity and sedentary bout metrics, including moderate-to-vigorous physical activity (MVPA), total physical activity, and sedentary behavior bouts.

c sedentary break metrics, reflecting interruptions in sedentary time.

d physical behavior summary metrics, including time spent in different intensity levels, step counts, and fragmentation indices.

e axis-specific acceleration counts (X, Y, Z) and vector magnitude (VM).

f sleep parameters derived using the ActiLife algorithm; and.

g additional formats, including percentage versions, alternative bout durations (e.g., 5 and 10 min), and SB classification using <100 cpm, based on vector magnitude, derived upon request from investigators and incorporated into the database as an alternative sedentary behavior metric. The primary sedentary behavior classification for the ELSA-Brasil database is based on the validated vector magnitude threshold of <200 cpm, as summarized in Table 3.

Wear-time compliance variables capture the number of valid monitoring days and total wear time, enabling objective verification of adherence to the accelerometer protocol. Activity and sedentary bout variables quantify the frequency and duration of sustained bouts of MVPA, total PA, and SB, based on validated cut-points and predefined bout criteria [1,18,19]. Sedentary break variables characterize the frequency and duration of interruptions in sedentary time, providing additional indicators of behavioral patterns independent of total sedentary exposure.

Awake physical behavior summary variables include daily estimates of time spent in SB, light, moderate, and vigorous intensity activities, total step counts, and activity fragmentation metrics, providing comprehensive characterization of activity volume, intensity distribution, and temporal variability. In addition, acceleration counts were preserved as both aggregated and minute-level summaries for each orthogonal axis (x, y, and z) and for the derived vector magnitude, ensuring retention of detailed movement information and enabling future re-analysis using alternative processing approaches.

Sleep variables were generated using standardized ActiLife sleep analysis configuration and include TST, the number of sleep periods, sleep efficiency, wake after sleep onset, sleep activity counts, and sleep fragmentation index. These parameters enable objective characterization of sleep duration, continuity, and quality under free-living conditions using validated accelerometry-based sleep detection methods [21,28,29].

The standardized implementation of this protocol, including information about device configuration, processing parameters, and software settings, ensures consistent generation of accelerometry-derived behavioral variables. The resulting dataset provides a structured analytical resource for objective assessment of PA, SB, and sleep in large epidemiological cohorts, and is consistent with methodological frameworks applied in other population-based accelerometry studies [1,6,31].

## Limitations

This accelerometer data processing protocol has some methodological limitations that should be considered. First, although hip placement is supported by evidence for accurately assessing PA and SB [17,34], the lack of sleep detection algorithms specifically developed and validated for hip-worn accelerometry may reduce sensitivity for detecting certain sleep parameters, particularly short awakenings, compared with wrist-worn devices [22]. While the Cole–Kripke algorithm was originally developed for wrist-worn accelerometry [28], its application to hip-worn accelerometry data in ELSA-Brasil is consistent with prior epidemiological studies but may introduce some bias in estimates of sleep fragmentation and related sleep metrics [21,36,37].

Second, the adoption of activity intensity cut-points validated for older adults, although methodologically justified given the age distribution of the cohort and the absence of cohort-specific calibration, may affect the precision of intensity classification, particularly among younger participants within the middle-aged subgroup [34]. Cut-point–based classification methods are inherently dependent on the population and calibration procedures used during their development, which may introduce some degree of misclassification when applied to heterogeneous populations.

Third, count-based activity metrics generated by ActiLife represent processed transformations of raw acceleration signals using proprietary filtering and processing algorithms. Therefore, exact reproduction of ActiLife-derived activity counts across different software versions, device firmware configurations, or independent processing platforms cannot be fully guaranteed, particularly when differences exist in signal filtering or processing implementation. In addition, count-based cut-point approaches are dependent on device model, firmware version, and signal processing implementation, which may limit direct comparability with raw acceleration–based processing methods, such as Euclidean Norm Minus One (ENMO), increasingly used in large-scale biobank-based studies and open-source processing frameworks [4,8,15,31]. However, reproducibility within the ActiLife ecosystem is supported when standardized device models, firmware versions, and software configurations are consistently applied.

Finally, although algorithm-based wear-time detection methods such as the Choi algorithm improve standardization and reduce reliance on self-reported logs [27,30], they remain indirect methods of wear-time classification and may introduce minor classification uncertainty in cases of prolonged inactivity or atypical movement patterns.

## Related research article

None.

## Declaration of competing interest

The authors declare that they have no known competing financial interests or personal relationships that could have appeared to influence the work reported in this paper.

## Data Availability

The authors do not have permission to share data.

## Appendix Supplementary materials

Supplementary material *and/or* additional information **Supplementary File 1.** ActiLife processing settings used in the ELSA-Brasil accelerometer data processing protocol. This file provides screenshots of the ActiLife configuration windows used for epoch conversion, filter configuration, wear-time validation, sleep analysis, and physical activity and sedentary behavior classification. Image, application 1
