# Supplementary material for: Accelerometer data processing pipeline using ActiLife software for reproducible 24-hour physical behavior assessment in the ELSA-Brasil cohort
Source: MethodsX. 2026 Aug 7;17:104092. doi: 10.1016/j.mex.2026.104092 (PMC13507815; doi:10.1016/j.mex.2026.104092)
Supplement: Supplementary file 1 [file mmc1.pdf]

MethodsX
Open access
Supplementary Material

Supplementary File 1

ActiLife processing settings used in the ELSA-Brasil accelerometer data processing protocol

Article information

Article title

Accelerometer data processing pipeline using ActiLife software for reproducible 24-hour physical behavior assessment in the ELSA-Brasil cohort.

Authors

Bruno Pereira de Moura[a]*, Jeffer Eidi Sasaki[b], Rosane Harter Griep[c], Natan Feter[d], Maria Inês Schmidt[e], Maria de Jesus Mendes da Fonseca[f]*

Affiliations

[a] brunoefi@gmail.com; https://orcid.org/0000-0002-8439-4523; Fundação Oswaldo Cruz. Escola Nacional de Saúde Pública Sérgio Arouca. Programa de Pós-graduação em Epidemiologia em Saúde Pública. Rio de Janeiro, RJ, Brazil.
[b] jesasaki@wisc.edu; https://orcid.org/0000-0002-2083-4104; University of Wisconsin–Madison. School of Education. Department of Kinesiology. Madison, WI, USA
[c] rohgriep@gmail.com; https://orcid.org/0000-0002-6250-2036; Fundação Oswaldo Cruz. Instituto Oswaldo Cruz. Laboratório de Educação em Ambiente e Saúde. Programa de Pós-graduação em Epidemiologia em Saúde Pública. Rio de Janeiro, RJ, Brazil
[d] feter@usc.edu; https://orcid.org/0000-0001-6295-9792; Postdoctoral Research Fellow, Department of Biological Sciences, University of Southern California, Los Angeles, USA.
[e] maria.schmidt@ufrgs.br; https://orcid.org/0000-0002-3837-0731; Postgraduate Program in Epidemiology and Hospital de Clínicas de Porto Alegre, Federal University of Rio Grande do Sul, Porto Alegre, Rio Grande do Sul, Brazil.
[f] maria.fonseca@fiocruz.br; https://orcid.org/0000-0002-5319-5513; Fundação Oswaldo Cruz. Escola Nacional de Saúde Pública Sérgio Arouca. Programa de Pós-graduação em Epidemiologia em Saúde Pública. Rio de Janeiro, RJ, Brazil.

Corresponding author's email address and Twitter handle

Bruno Pereira de Moura: brunoefi@gmail.com
Maria de Jesus Mendes da Fonseca: maria.fonseca@fiocruz.br

Cite this dataset

Moura, Bruno (2026), "Supplementary File 1: ActiLife processing settings used in the ELSA-Brasil accelerometer data processing protocol", Mendeley Data, V1, doi: 10.17632/7rvp458m48.1

MethodsX
Open access
Supplementary Material

1. Background

This supplementary file documents the ActiLife software settings used to process accelerometer data in the ELSA-Brasil protocol. The screenshots show the software version, raw-to-epoch conversion settings, wear-time validation parameters, sleep analysis configuration, and physical activity and sedentary behavior classification settings. These materials are provided to improve transparency, support replication of the processing workflow, and clarify the exact configuration used to generate the ActiLife-derived variables described in the main manuscript.

2. Accelerometer model used in the ELSA-Brasil cohort

The ELSA-Brasil accelerometry protocol used the ActiGraph wGT3X-BT device for 24-hour hip-worn monitoring under free-living conditions.

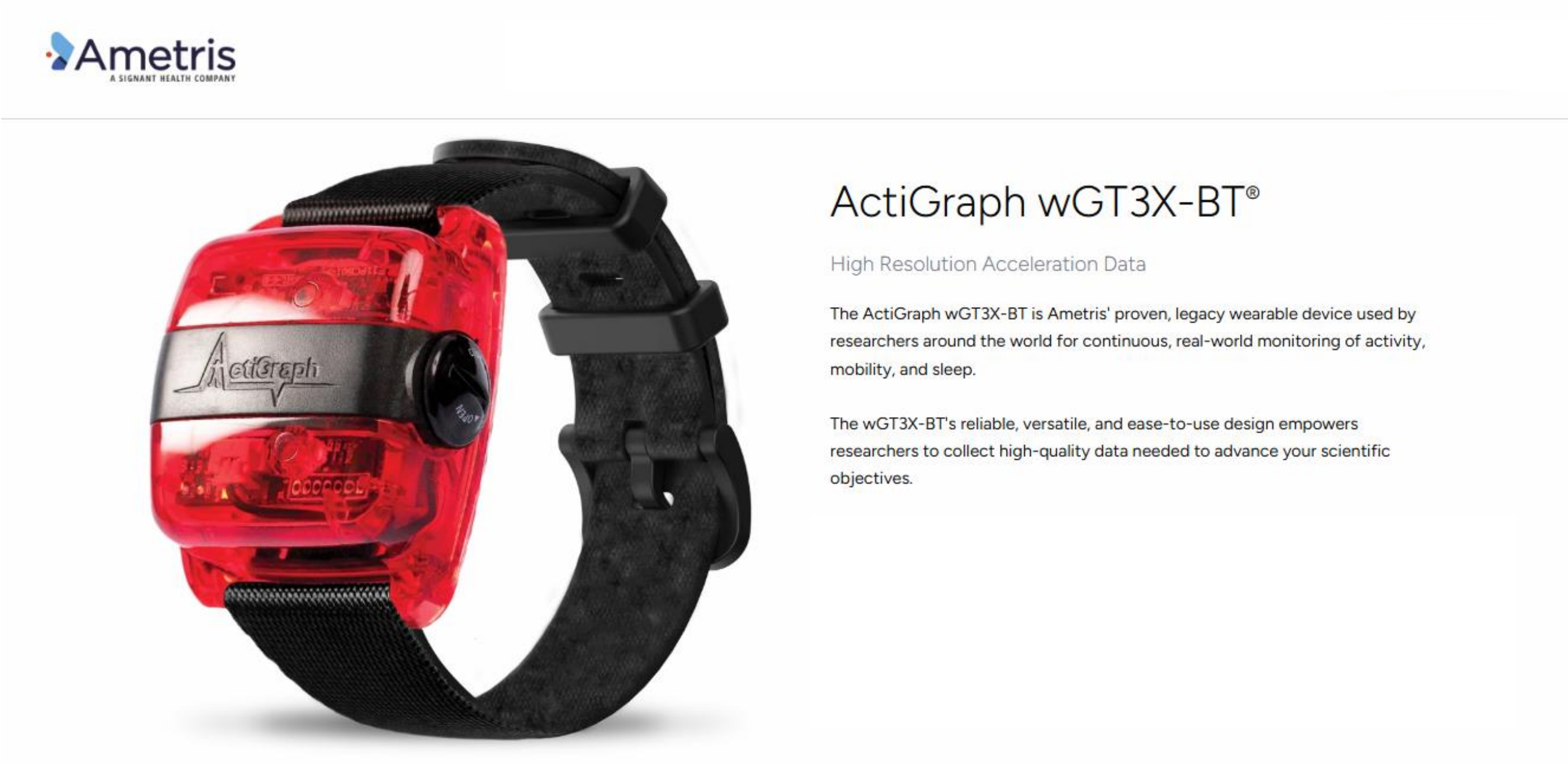

Figure S1. ActiGraph wGT3X-BT accelerometer used in the ELSA-Brasil accelerometry protocol. The device was worn on the right hip for seven consecutive days and used to collect raw triaxial acceleration data under free-living conditions. Source: Ametris/ActiGraph product page, https://ametris.com/actigraph-wgt3x-bt.

3. ActiLife software version

ActiLife version 6.13.5 was used for all accelerometer data processing steps in this supplementary file.

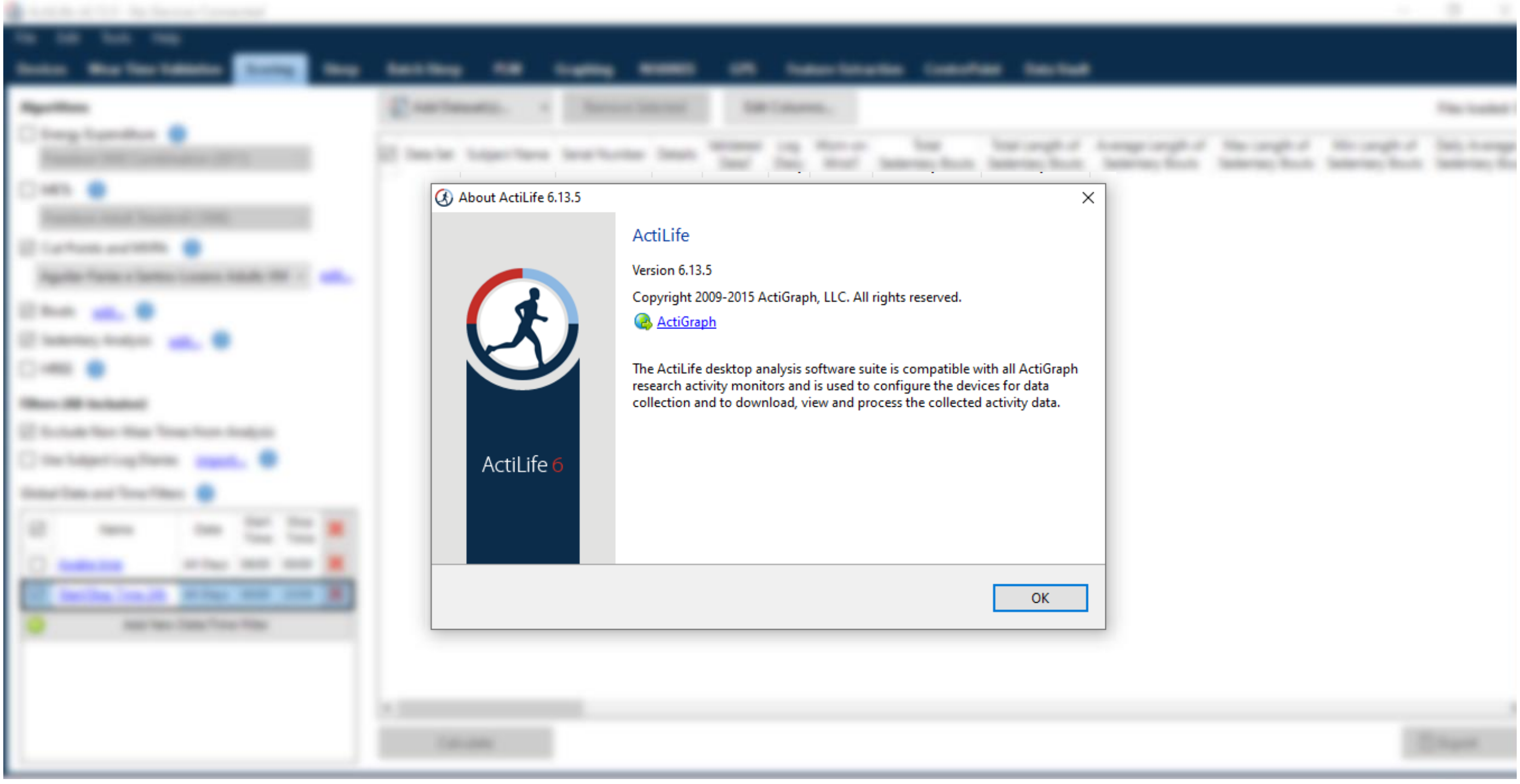

Figure S2. ActiLife software version used for accelerometer data processing. All accelerometer files were processed using ActiLife version 6.13.5 to ensure consistency across the generation of epoch-level files, wear-time validation, sleep analysis, and physical activity/sedentary behavior classification.

4. Conversion of raw accelerometer data to epoch format

Raw .gt3x files were converted to epoch-level .agd files using the Import/Export/Convert function in ActiLife.

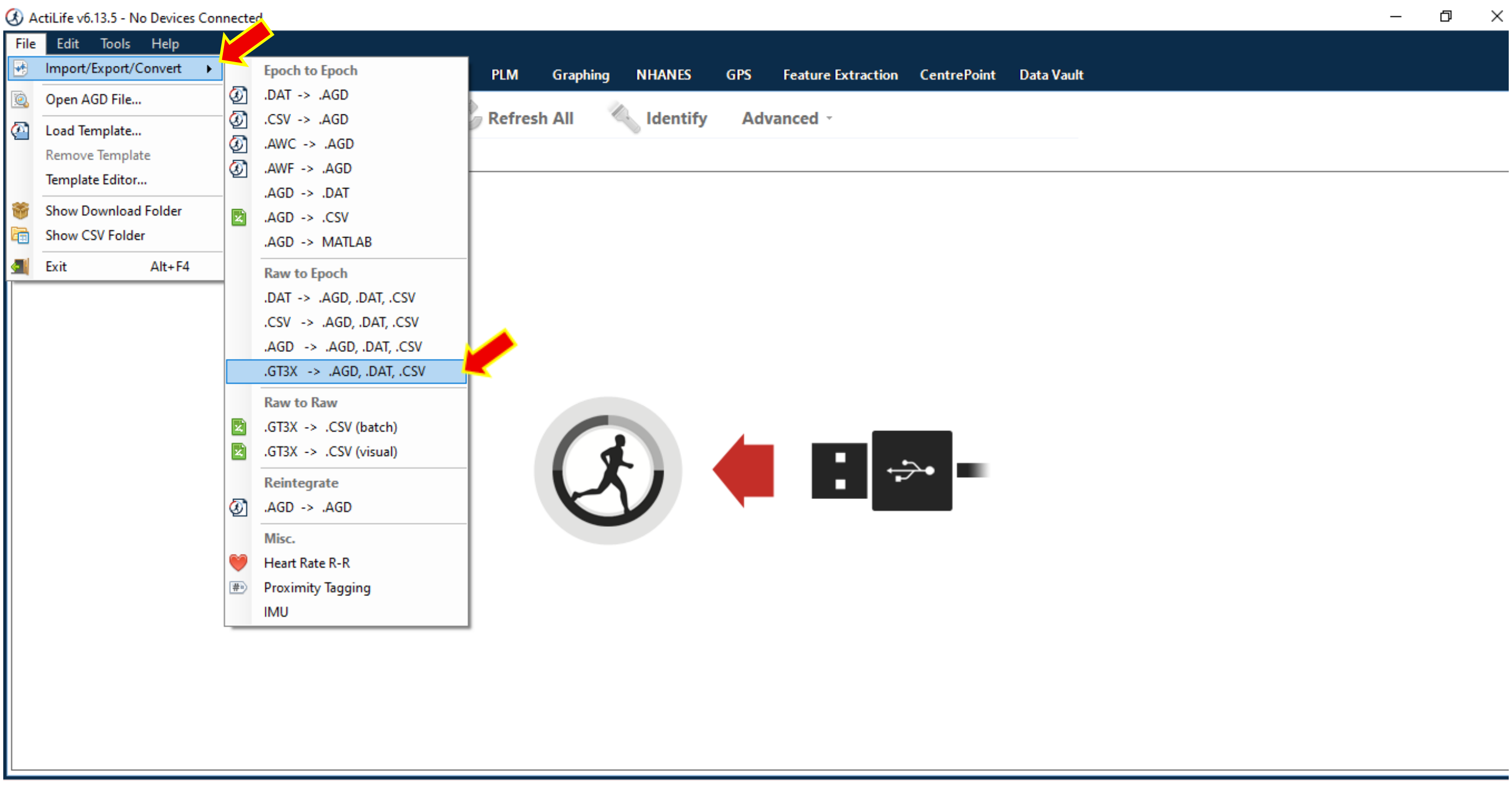

Figure S3. ActiLife menu pathway used for raw-to-epoch conversion. The Import/Export/Convert function was used to convert raw .gt3x accelerometer files into epoch-level .agd files for subsequent analysis.

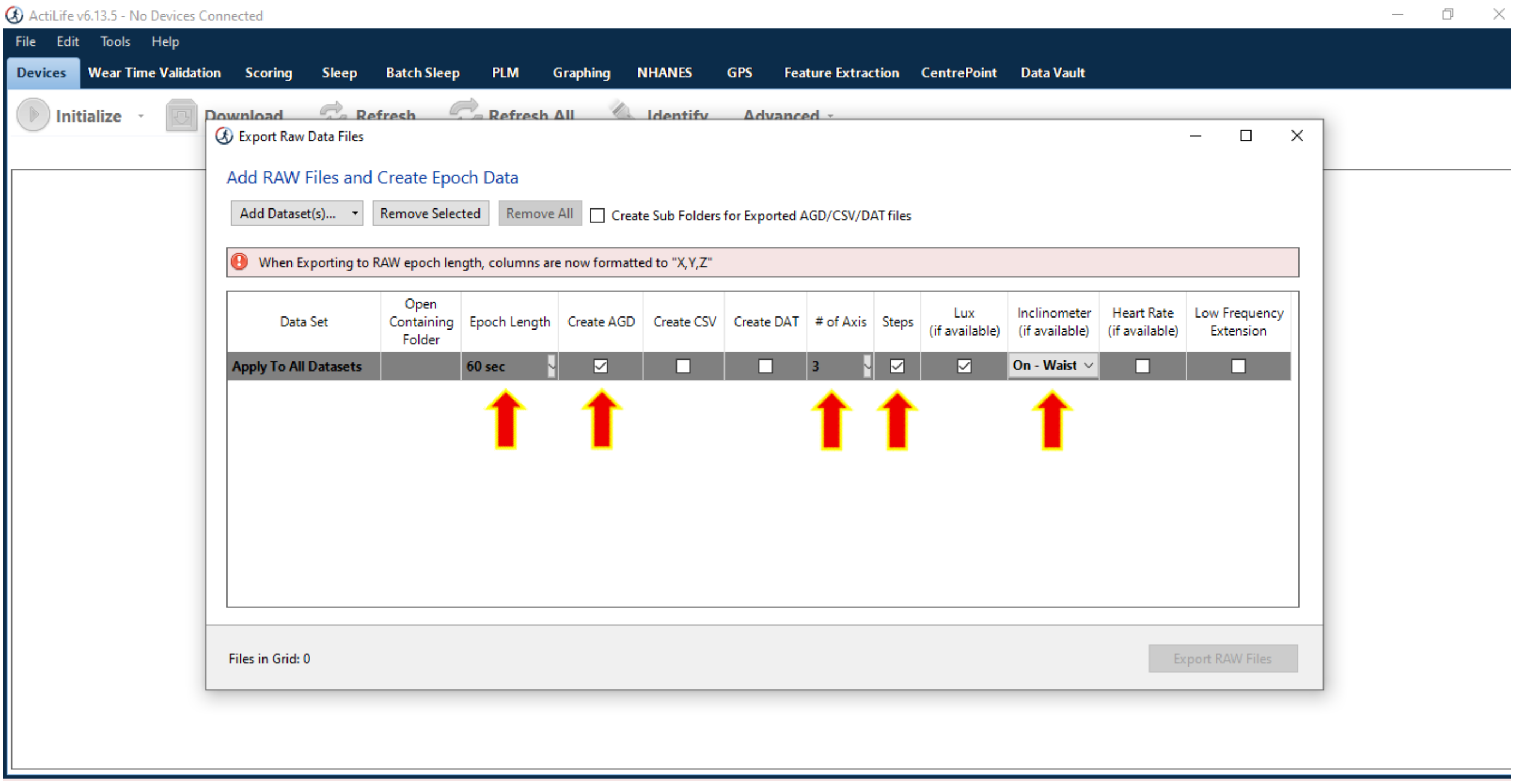

Figure S4. Raw-to-epoch conversion settings in ActiLife. Raw accelerometer files were converted using 60-second epochs, with steps and inclinometer data retained, the Normal filter enabled, and Low Frequency Extension disabled.

5. Wear time validation

Wear and non-wear periods were identified using the Choi algorithm implemented within the ActiLife Wear Time Validation tab.

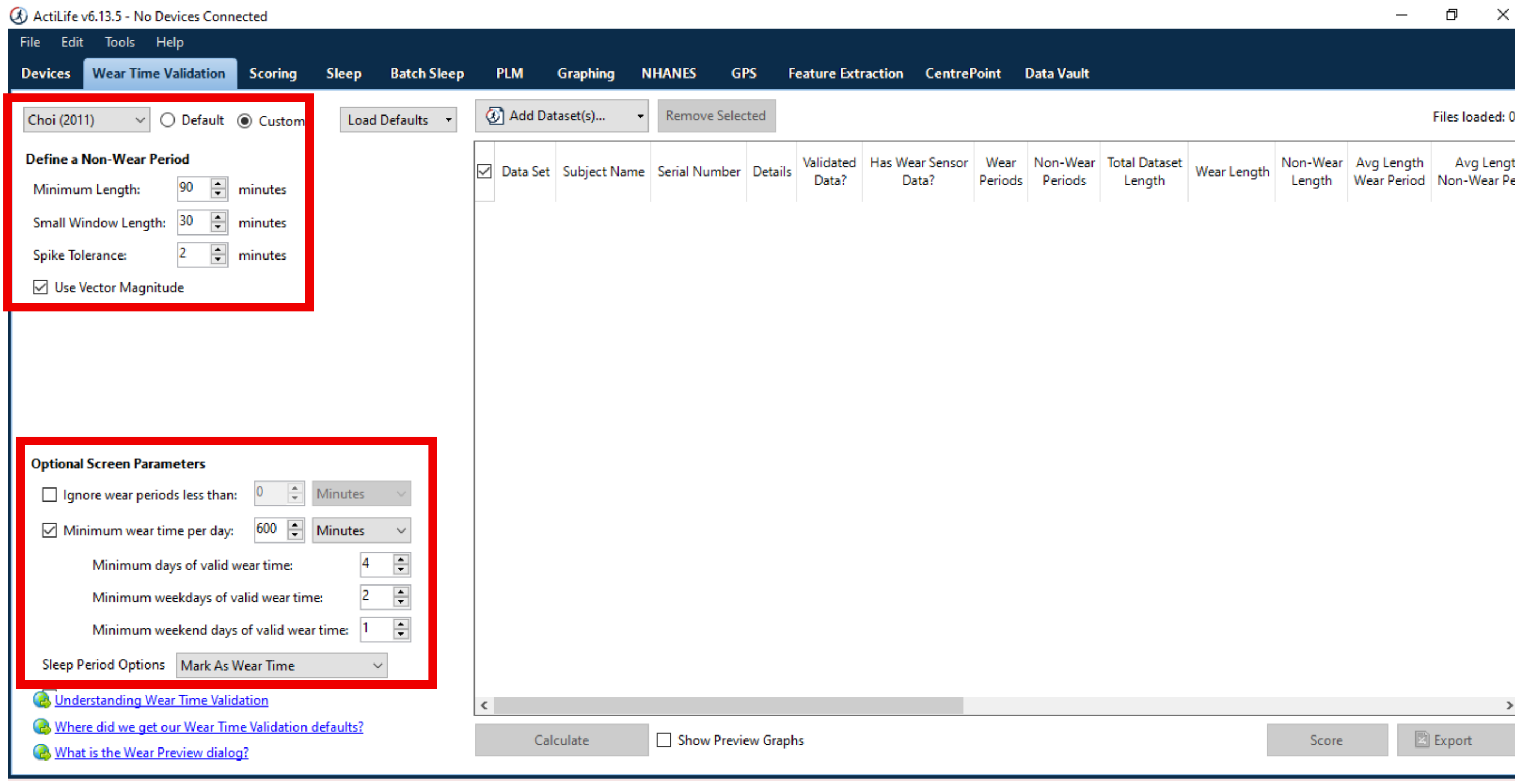

Figure S5. Wear-time validation settings. Non-wear time was identified using the Choi algorithm with a minimum non-wear duration of 90 minutes, a 2-minute spike tolerance, and a 30-minute upstream/downstream window. Vector magnitude was selected as the primary input. A valid day was defined as ≥600 minutes of wear time, with at least four valid days required, including at least two weekdays and one weekend day. Sleep periods were marked as wear time.

6. Sleep analysis

Sleep was analyzed using the ActiLife Batch Sleep function, combining the Cole–Kripke sleep scoring algorithm with the Tudor–Locke sleep-period detection algorithm.

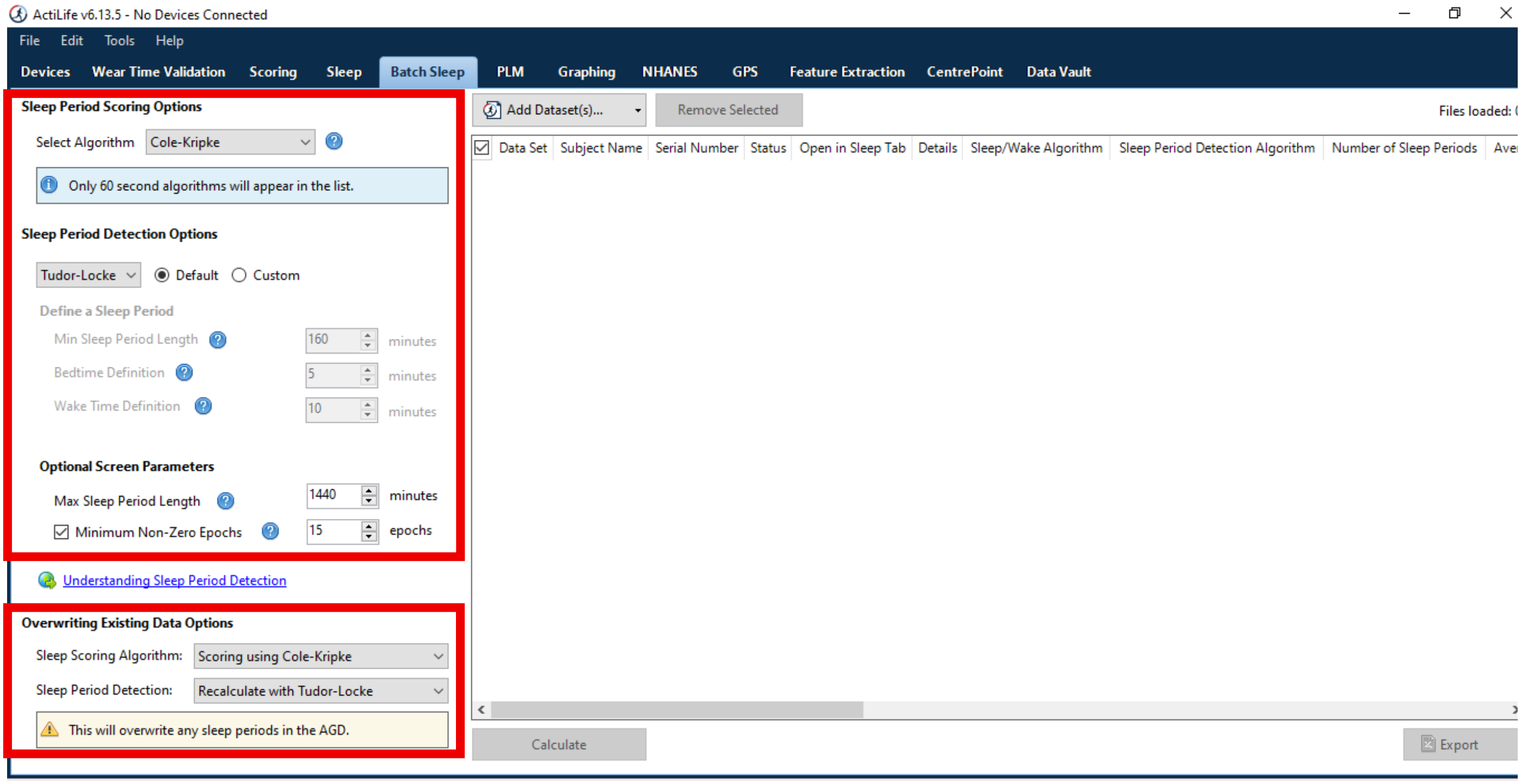

Figure S6. Sleep analysis configuration in ActiLife. The Batch Sleep function was configured using the Cole–Kripke sleep scoring algorithm and the Tudor–Locke sleep-period detection algorithm. Default detection parameters were applied, including a minimum sleep period of 160 minutes, a maximum sleep period of 1,440 minutes, and a minimum of 15 non-zero epochs.

MethodsX
Open access
Supplementary Material

7. Physical activity and sedentary behavior classification

Waking physical activity and sedentary behavior were classified using vector magnitude cut-points in the ActiLife Scoring tab.

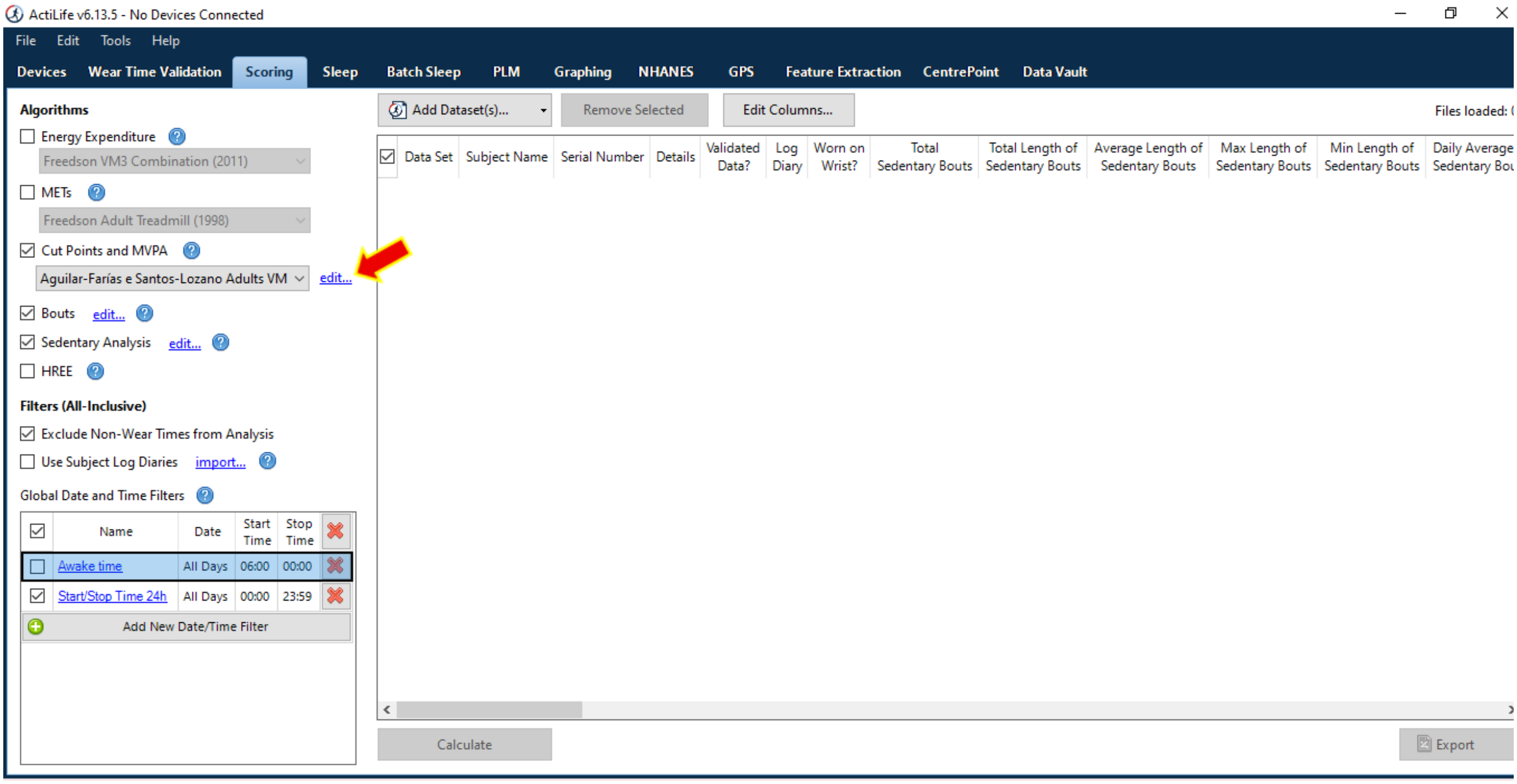

Figure S7. ActiLife scoring interface for physical activity and sedentary behavior classification. The Scoring tab was used to apply vector magnitude cut-points, calculate bouts, perform sedentary analysis, exclude non-wear time from analysis, and define global date/time filters.

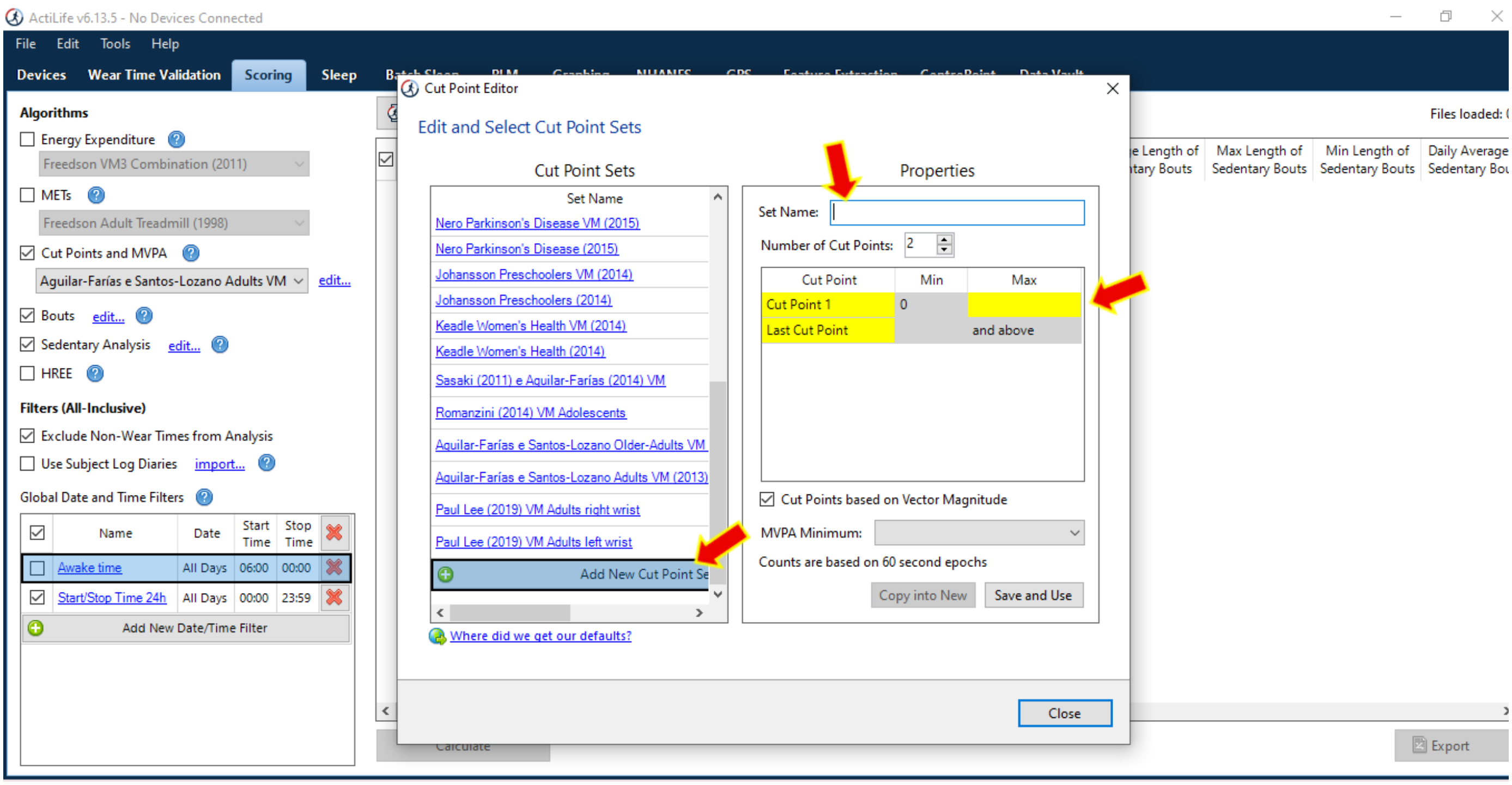

Figure S8. Custom cut-point editor used to define vector magnitude intensity thresholds. A custom cut-point set was created in ActiLife to classify sedentary behavior, light-intensity physical activity, moderate-intensity physical activity, and vigorous-intensity physical activity using vector magnitude counts per minute.

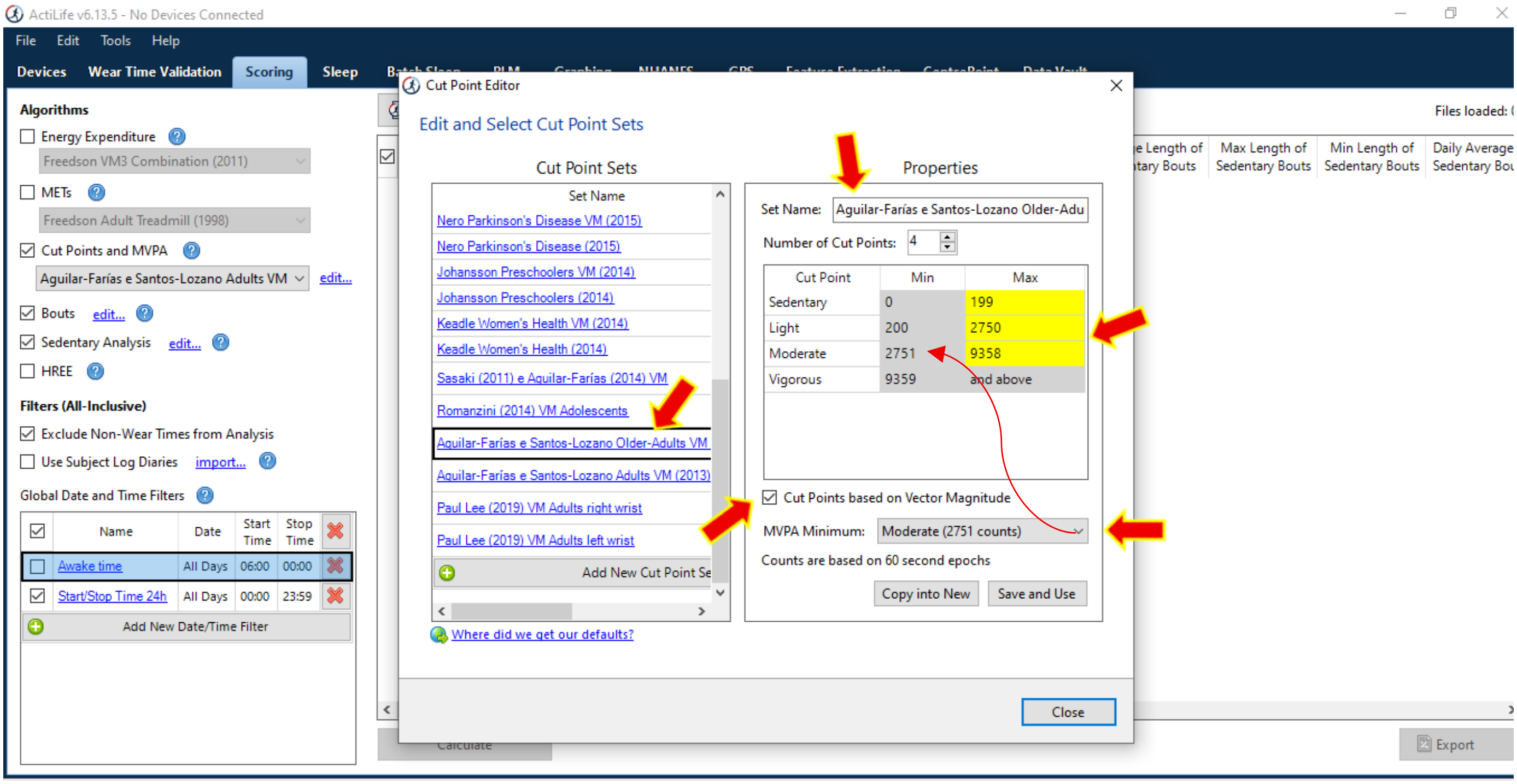

Figure S9. Vector magnitude cut-points used for intensity classification. Sedentary behavior was defined as 0–199 counts per minute, light-intensity physical activity as 200–2,750 counts per minute, moderate-intensity physical activity as 2,751–9,358 counts per minute, and vigorous-intensity physical activity as ≥9,359 counts per minute. Moderate-to-vigorous physical activity was defined as ≥2,751 counts per minute.

The subsequent screenshots document the bout definitions and sedentary behavior settings applied in the ActiLife Scoring tab.

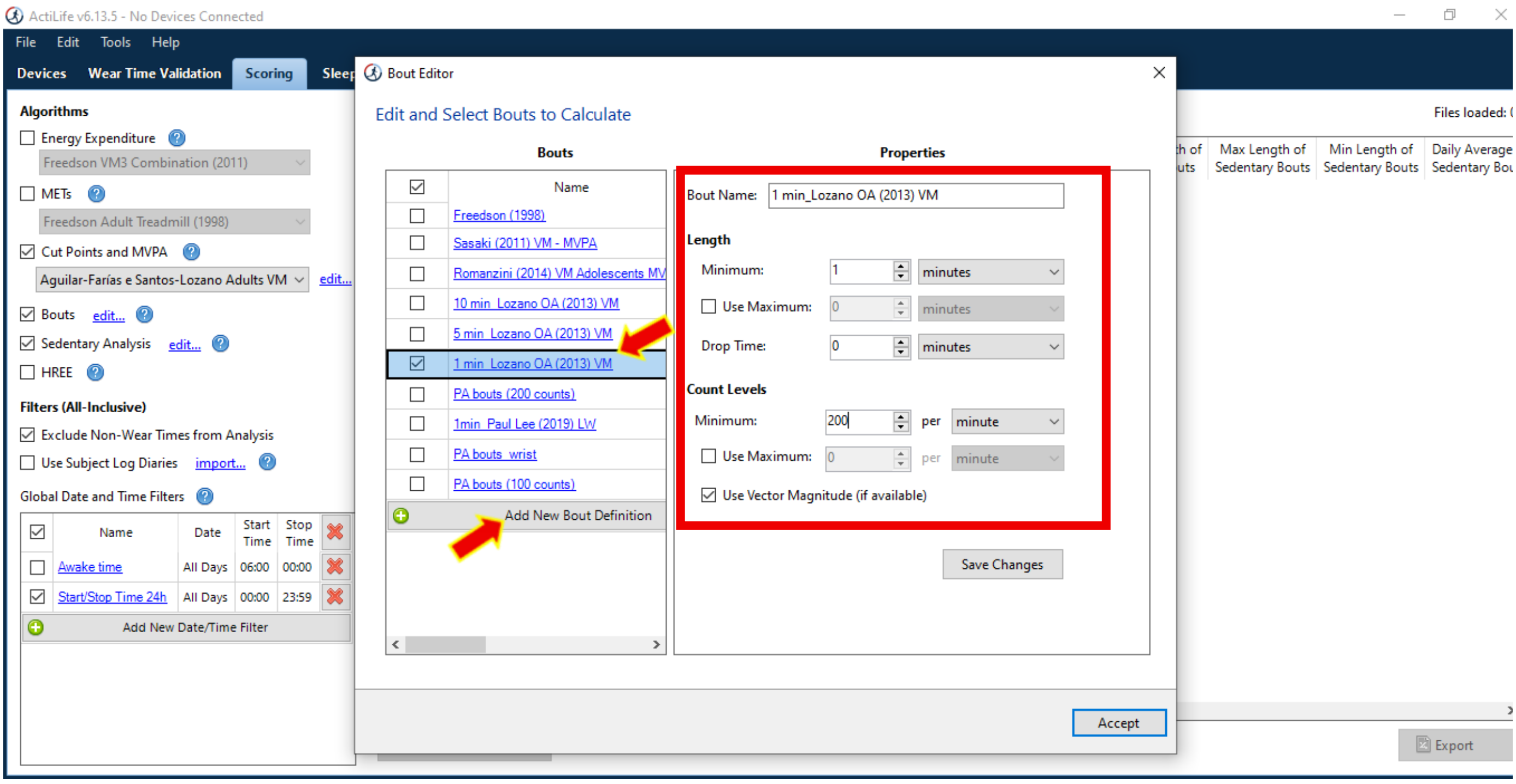

Figure S10. Bout definition for total physical activity. Total physical activity bouts were defined using vector magnitude counts ≥200 counts per minute, with a minimum bout duration of 1 minute and a drop time of 0 minutes.

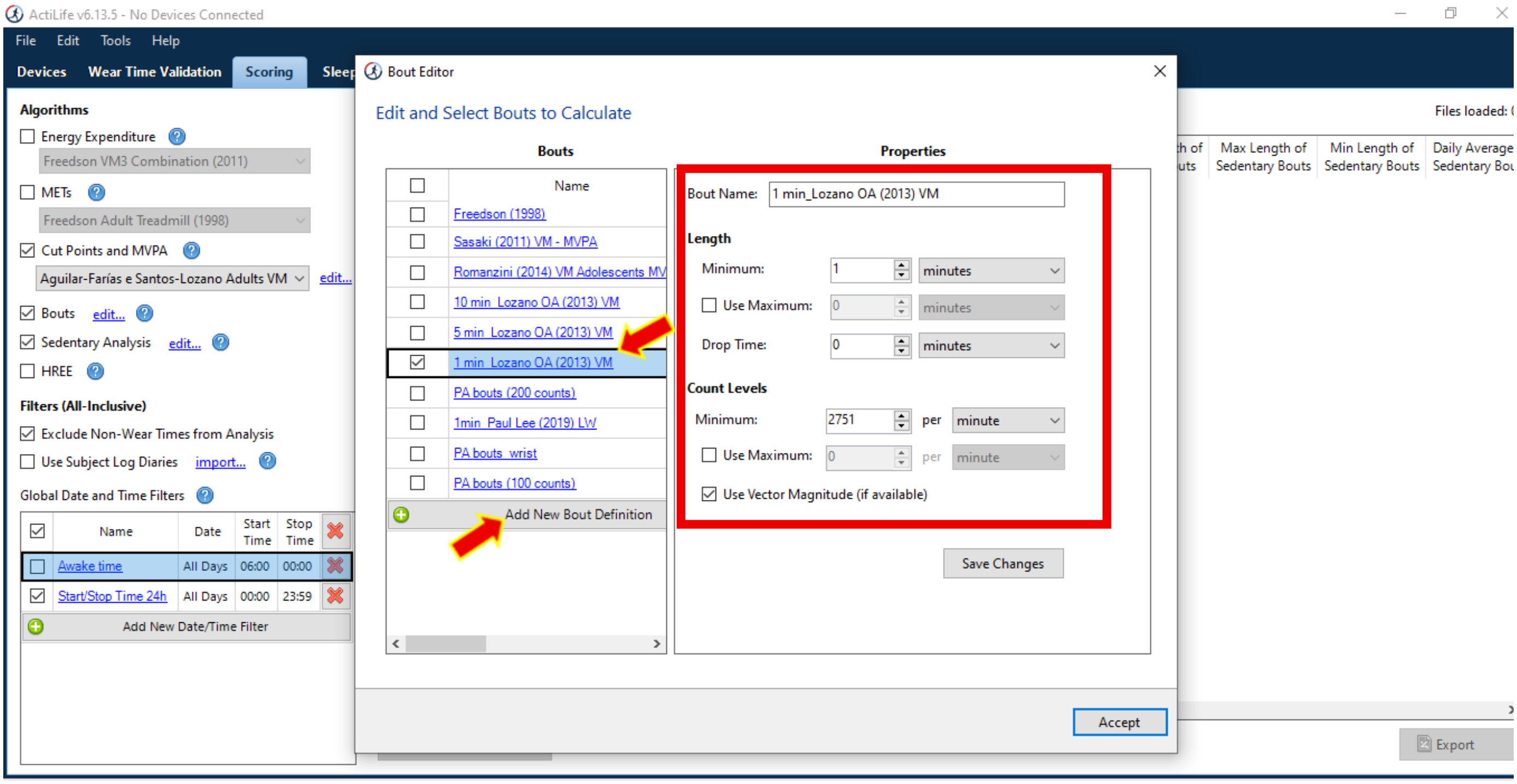

Figure S11. Bout definition for moderate-to-vigorous physical activity. MVPA bouts were defined using vector magnitude counts ≥2,751 counts per minute, with a minimum bout duration of 1 minute and a drop time of 0 minutes.

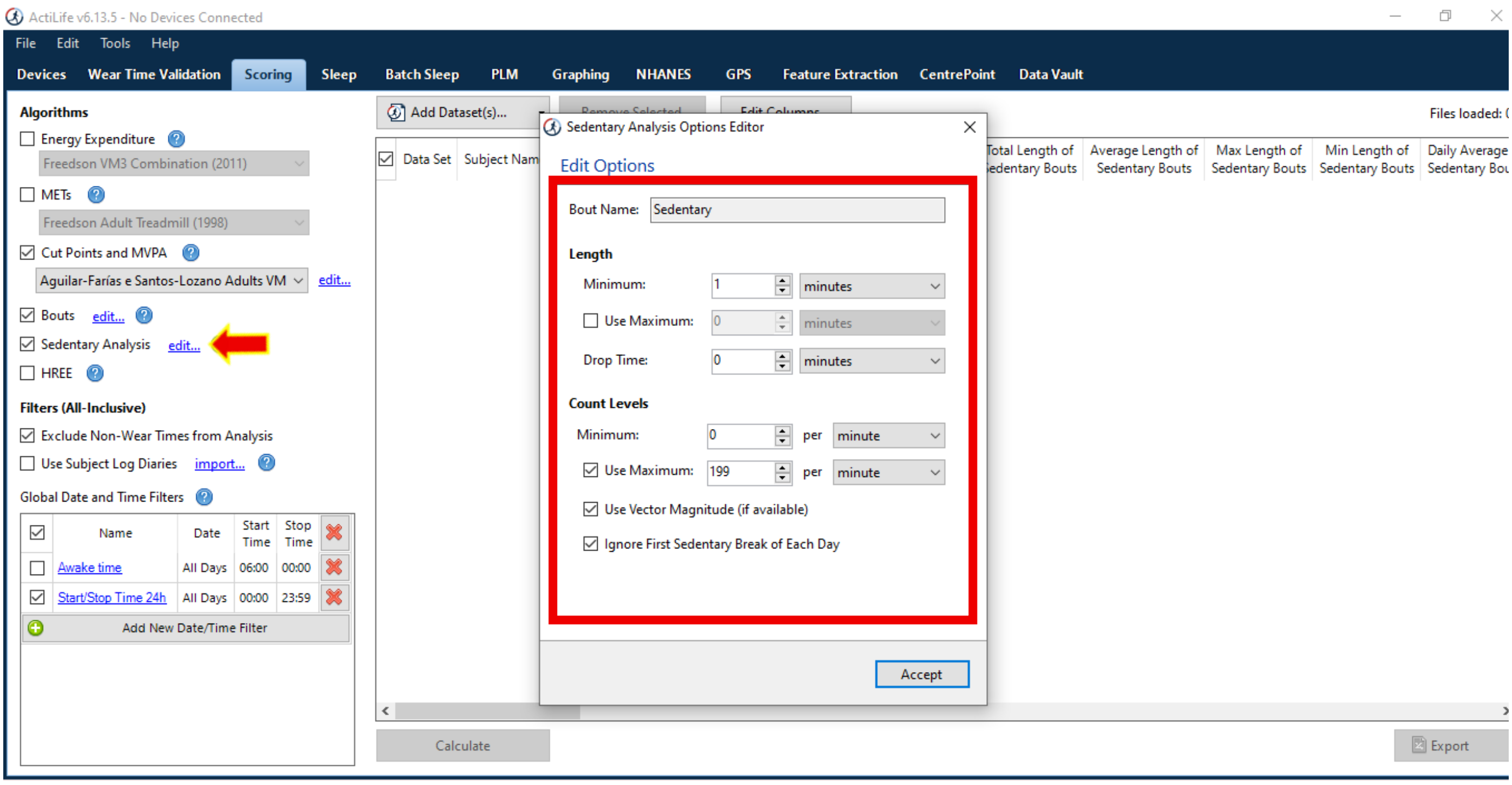

Figure S12. Sedentary behavior bout settings. Sedentary behavior bouts were defined using vector magnitude counts below 200 counts per minute, with a minimum duration of 1 minute and a drop time of 0 minutes. The first sedentary break of each day was ignored according to the ActiLife sedentary analysis configuration.

8. Global date and time filters

Global date and time filters were configured to define the daily analysis windows used for waking behavior and 24-hour physical behavior summaries.

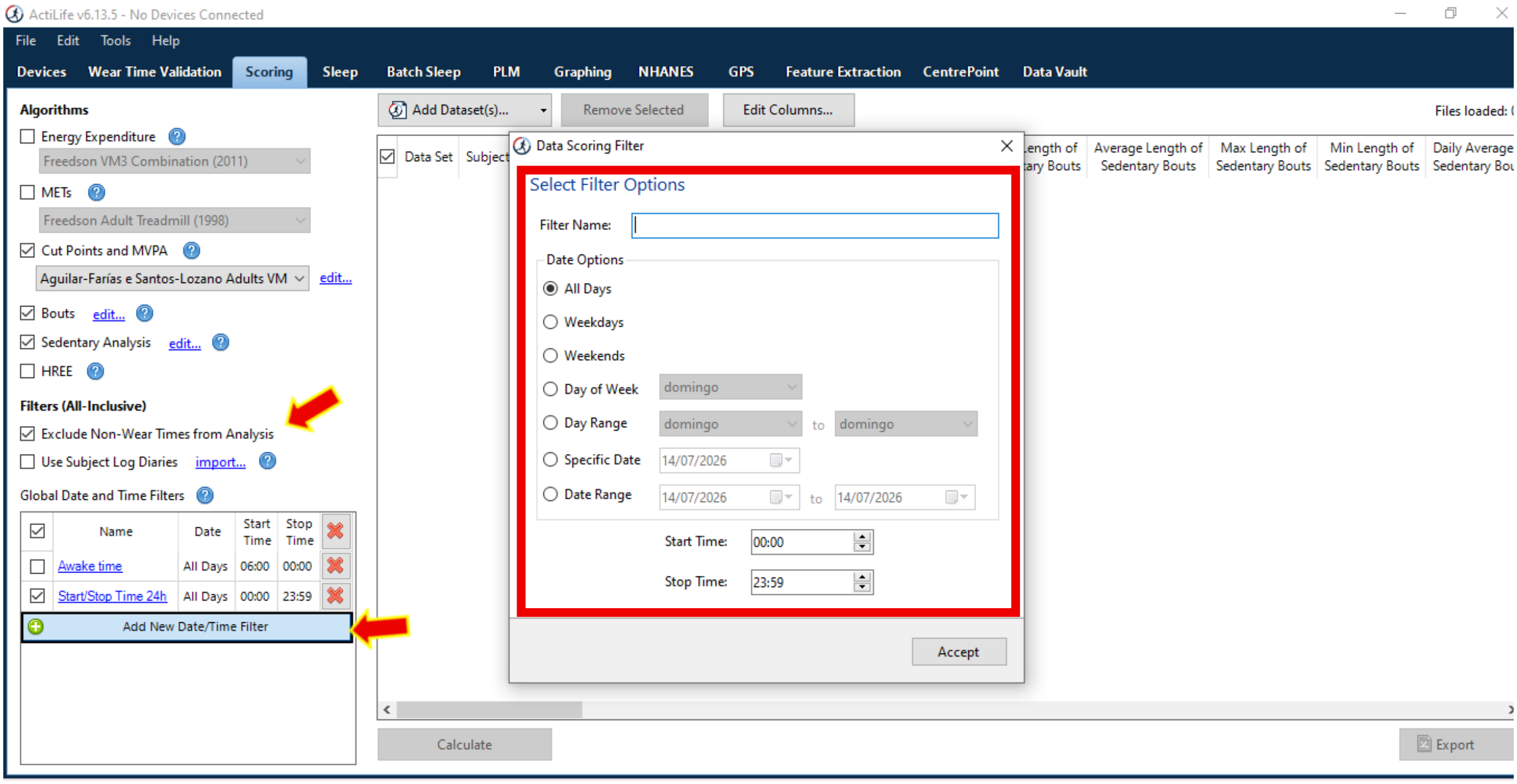

Figure S13. Global date and time filter for waking behavior analysis. The Awake Time filter was configured to include all days from 00:00 to 23:59 and was used for analysis of waking physical activity and sedentary behavior variables.

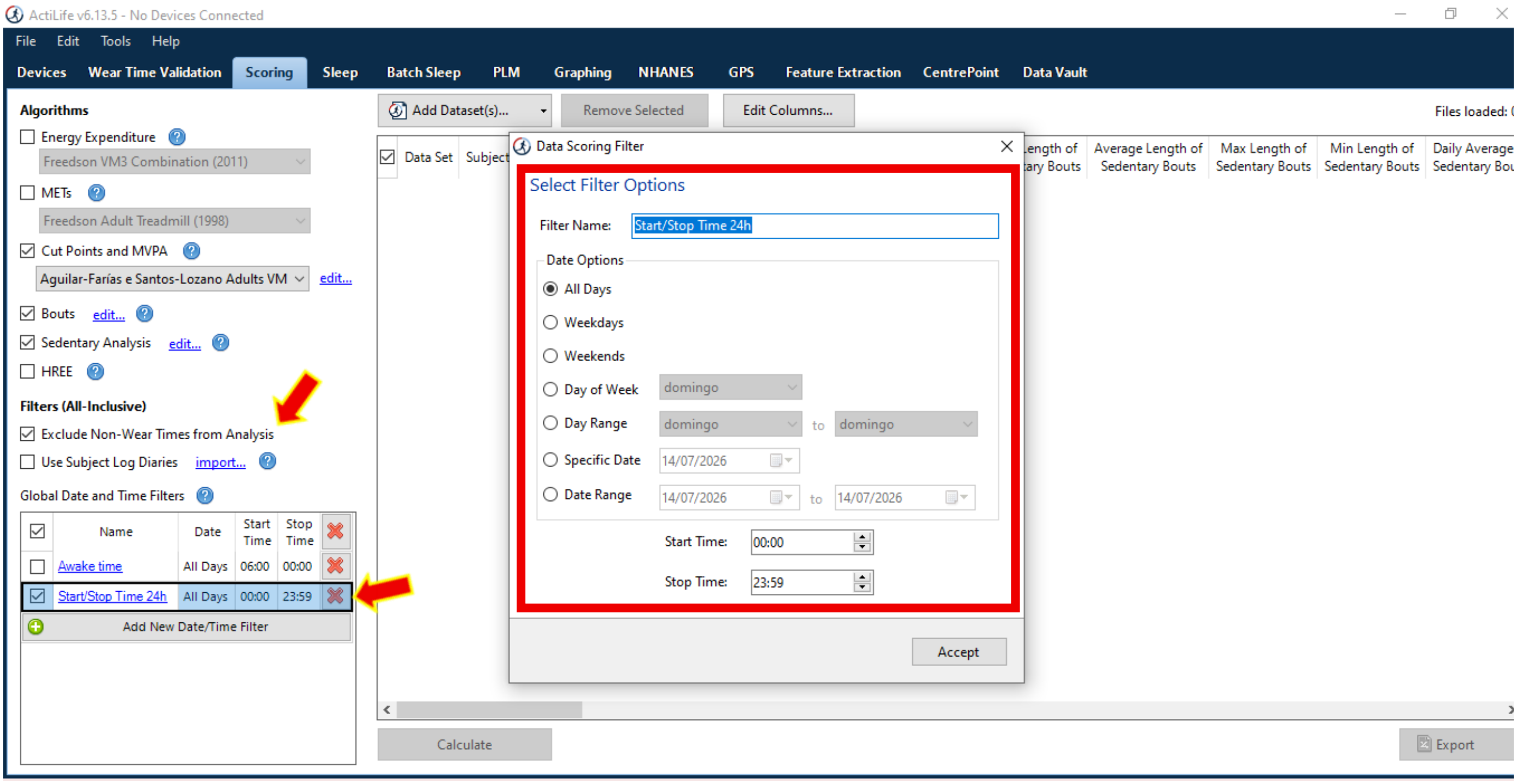

Figure S14. Global date and time filter for 24-hour physical behavior analysis. The 24-hour Physical Time filter was configured to include all days from 00:00 to 23:59, supporting standardized daily estimates across valid monitoring days.

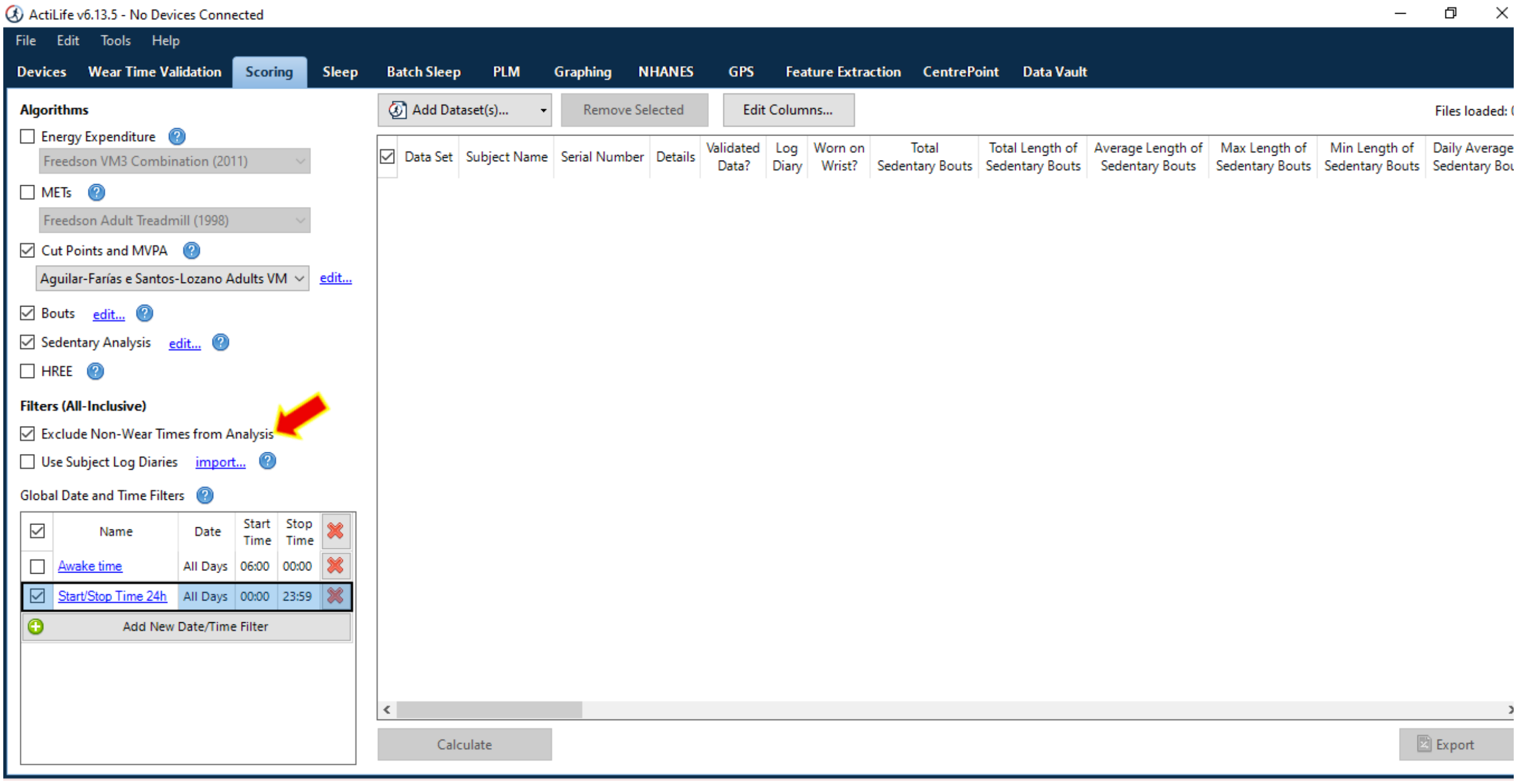

Figure S15. Non-wear exclusion and participant activity log settings. Non-wear periods were excluded from analysis, and participant activity logs were not used for physical activity and sedentary behavior classification. These settings supported standardized, algorithm-based processing of waking behavior variables.

Cite this dataset

Moura, Bruno (2026), "Supplementary File 1: ActiLife processing settings used in the ELSA-Brasil accelerometer data processing protocol", Mendeley Data, V1, doi: 10.17632/7rvp458m48.1
